# Risk of Infections With Infliximab vs Adalimumab Among Children With Inflammatory Bowel Disease

**DOI:** 10.1001/jamanetworkopen.2026.22684

**Published:** 2026-07-10

**Authors:** Ning Lyu, Michaela Tracy, Sebastian Schneeweiss, Timothy J. Savage

**Affiliations:** 1 Division of Pharmacoepidemiology and Pharmacoeconomics, Department of Medicine, Brigham and Women’s Hospital, Boston, Massachusetts; 2 Harvard-MIT Center for Regulatory Science, Harvard Medical School, Boston, Massachusetts; 3 Division of Gastroenterology, Hepatology, and Nutrition, Boston Children’s Hospital, Boston, Massachusetts; 4 Department of Pediatrics, Harvard Medical School, Boston, Massachusetts; 5 Department of Medicine, Harvard Medical School, Boston, Massachusetts; 6 Division of Infectious Diseases, Boston Children’s Hospital, Boston, Massachusetts

## Abstract

**Importance:**

Infliximab and adalimumab are the only biologics approved by the US Food and Drug Administration for use in children with inflammatory bowel disease (IBD). The comparative infection risks between infliximab and adalimumab have not been evaluated in clinical settings.

**Objective:**

To compare the risk of infections with infliximab vs adalimumab among pediatric patients with IBD using nationwide claims data.

**Design, Setting, and Participants:**

This cohort study used data from 2 nationwide health care claims databases from January 1, 2016, to February 28, 2025. The study included children and adolescents aged 6 to 17 years with a diagnosis of Crohn disease or ulcerative colitis who newly initiated infliximab or adalimumab and had 180 days or more of prior continuous enrollment.

**Exposures:**

Initiation of adalimumab vs infliximab.

**Main Outcomes and Measures:**

Serious infections were defined as infections requiring hospitalization, using a validated algorithm. Outpatient infections were defined as an outpatient infection diagnosis with a targeted antimicrobial dispensed within 1 day. Baseline covariates within each dataset were balanced with 1:1 propensity score matching and results were pooled using fixed effects meta-analysis. Incidence rates and hazard ratios (HRs) were estimated over a 180-day follow-up.

**Results:**

After applying exclusion criteria, 4239 children with IBD initiating infliximab (2467; mean [SD] age, 13.3 [2.9] years; 1462 boys [59.3%]; 1502 with Crohn disease [60.9%]) or adalimumab (1772; mean [SD] age, 14.0 [2.7] years; 1047 boys [59.1%]; 1068 with Crohn disease [60.3%]) were identified. After matching, each exposure group included 1533 patients. The incidence of serious infections was 29 per 1000 person-years for infliximab (reference) and 34 per 1000 person-years for adalimumab (pooled HR, 1.15; 95% CI, 0.63-2.11). Outpatient infections were more common, with rates of 358 per 1000 person-years for infliximab and 386 per 1000 person-years for adalimumab (pooled HR, 1.08; 95% CI, 0.90-1.29). Results of sensitivity analyses, including restriction to monotherapy, originator-only use, first-line users only, exclusion of gastrointestinal infections from the outcome, and extending follow-up to 365 days, were all consistent with the primary findings. No mycobacterial infections were observed in the overall cohort.

**Conclusions and Relevance:**

In this cohort study of children and adolescents with IBD, serious infections were rare. No evidence of differences in infection risk were observed between infliximab and adalimumab. These findings suggest that the 2 agents may have comparable infection risk profiles in pediatric IBD, although meaningful differences cannot be ruled out.

## Key Points

**Question:**

Are there differences in the risk of serious infections or outpatient infections between infliximab and adalimumab for children and adolescents with inflammatory bowel disease (IBD)?

**Findings:**

In this cohort study of 4239 pediatric patients with IBD prescribed infliximab or adalimumab, serious infections were rare and outpatient infections were more frequent. There was no evidence of a difference in the risk of either serious or outpatient infections between the 2 agents.

**Meaning:**

These findings suggest that infliximab and adalimumab may have comparable infection risk profiles in pediatric IBD, but cannot rule out meaningful differences.

## Teaser

This cohort study uses nationwide claims data to examine the risk of serious infections and outpatient infections with infliximab vs adalimumab among children and adolescents with inflammatory bowel disease (IBD).

## Introduction

The prevalence of pediatric inflammatory bowel disease (IBD)—including Crohn disease (CD), ulcerative colitis (UC), and IBD-unclassified (IBD-U)—is increasing in the US, affecting an estimated 122 per 100 000 children.^1^ Since the approval of infliximab to treat IBD in 1998, anti–tumor necrosis factor (TNF) therapies have changed the treatment landscape of pediatric IBD. Infliximab and adalimumab were the first biologics approved by the US Food and Drug Administration for children with IBD. Among individuals with IBD, 43% received biologic therapy before 18 years of age, reflecting the increased use of the agents among children and highlighting the need for general pediatricians to understand the potential risks of these medications.^2^

Randomized clinical trials (RCTs) with sample sizes ranging from 60 to 188 have shown that both agents are effective in children with IBD; however, their comparative safety, particularly the risk of infections, remains unclear because no head-to-head trials have been conducted.^3,4,5,6^ Infections are an important safety concern in children with IBD, as population-based data suggest that children with IBD have a substantially higher rate of serious infection than children in the general population.^7^ However, these studies did not address the comparative safety of infliximab and adalimumab and outpatient infections have been even less well studied in children. Although several adult studies have compared the risk of serious infections between infliximab and adalimumab, these findings are inconsistent and may not be generalizable to pediatric patients.^8,9,10^

Although current guidelines recommend both infliximab and adalimumab as first-line options for pediatric CD and UC, robust comparative safety data could guide treatment choice.^11,12^ To address this evidence gap, this study aimed to compare the risk of infections with adalimumab vs infliximab among pediatric patients with IBD in routine clinical practice.

## Methods

### Data Source

We used 2 nationwide US commercial claims databases: Merative MarketScan Commercial Database (January 1, 2016, to December 31, 2023) and Optum’s deidentified Clinformatics Data Mart Database (January 1, 2016, to February 28, 2025). Both databases contain longitudinal information on enrollment, demographics, pharmacy dispensing, and diagnoses. Due to the deidentified nature of the data, the Brigham and Women’s Hospital institutional review board determined this study was not human participants research and therefore exempt from the requirement for informed consent. This study followed the Strengthening the Reporting of Observational Studies in Epidemiology (STROBE) reporting guideline.^13^

### Study Population

We defined the cohort entry date as the first dispensation or administration date of adalimumab (exposure group) or infliximab (reference group) (eFigure in Supplement 1). Medication use was identified from outpatient pharmacy claims using generic names and from outpatient and inpatient medical claims using Healthcare Common Procedure Coding System codes (eTable 1 in Supplement 1). Patients without an *International Statistical Classification of Diseases and Related Health Problems, Tenth Revision* (*ICD-10*), code for CD (K50.xx) or UC (K51.xx) within 365 days prior to cohort entry were excluded. Patients with diagnosis codes for both CD and UC were defined as IBD-U. This definition, requiring at least 1 IBD diagnosis code and 1 prescription claim for infliximab or adalimumab, was chosen to improve specificity to at least 85% beyond diagnosis codes alone, consistent with prior validation literature.^14^ We excluded patients younger than 6 years (to exclude very early-onset IBD) or older than 17 years at cohort entry. We ensured a new-user design through excluding patients with prior use of any biologics to treat IBD (infliximab, adalimumab, certolizumab pegol, natalizumab, vedolizumab, ustekinumab, risankizumab, or golimumab) in the 180 days prior to cohort entry.

Patients were excluded if they had fewer than 180 days of continuous enrollment, allowing a 30-day gap in medical or pharmacy benefits, before cohort entry or had HIV, congenital immunodeficiency, organ transplant, cancer, or pregnancy. We excluded patients with other indications for the study medications (rheumatoid arthritis, juvenile idiopathic arthritis, psoriatic arthritis, psoriasis, or ankylosing spondylitis). Patients exposed to both drugs on the cohort entry date were excluded. For patients with multiple qualifying biologic initiations, only the first event was included (eTable 2 in Supplement 1).

### Outcome

Outcomes were serious infections requiring hospitalization and outpatient infections requiring treatment. Serious infections were defined as a hospitalization with an *ICD-10* code in any position for meningitis, osteomyelitis, bacteremia, pneumonia, pyelonephritis, serious gastrointestinal infections, or skin and soft tissue infections (eTable 3 in Supplement 1), a definition with a previously validated positive predictive value of 80.2% for confirmed serious infection events.^15^ Outpatient infections were defined as an *ICD-10* code for infection (bacterial, mycobacterial, yeast, or viral) plus a dispensation of an appropriate antimicrobial within 1 day (eTable 3 in Supplement 1).^16,17,18^ This narrow window was selected to strengthen the temporal linkage between diagnosis and treatment and improve specificity of the outcome definition.

Follow-up began 1 day after cohort entry and continued until the earliest occurrence of the outcome, death, disenrollment, discontinuation of the index treatment, crossover to the comparator treatment, end of data availability, or 180 days after cohort entry. Discontinuation or switching of the index drug was defined using a grace period equal to 1.5 times the typical dosing interval.^19^ After the final observed infliximab administration, follow-up remained exposed for 56 days, and discontinuation was defined by a gap of more than 84 days. After the final observed adalimumab dispensing, follow-up remained exposed for 28 days, and discontinuation was defined by a gap of more than 42 days.

### Pretreatment Patient Characteristics

Demographic characteristics included age categories at cohort entry, sex, geographic region, and insurance type (available only in MarketScan). Clinical characteristics included IBD subtype (CD, UC, or IBD-U) and markers of IBD activity (esophagoduodenoscopy, colonoscopy or sigmoidoscopy, computed tomography of the abdomen or pelvis, magnetic resonance enterography, gastrointestinal bleeding, abnormal weight loss, and complicated IBD). Baseline comorbidities were captured using the Pediatric Comorbidity Index^20^ and select chronic conditions (chronic kidney disease, chronic liver disease, asthma, and anemia). Medication included prior use (180 to 31 days before cohort entry) and concurrent use (within 30 days of and including cohort entry) of thiopurines, methotrexate, and aminosalicylates. Cumulative systemic oral corticosteroid use in the 60 days before cohort entry date was quantified in prednisone-equivalent doses.^21^ Antibiotic, antiviral, and antifungal use was assessed as proxies of infection risk. Receipt of influenza and pneumococcal (extended) vaccines within the 365 days prior to cohort entry were assessed as a marker of preventive care engagement. Healthcare utilization was characterized by the number of emergency department visits, hospitalizations, and outpatient visits during the preceding 180 days. Unless otherwise noted, covariates were assessed during the 180 days before cohort entry. A complete list of baseline characteristics is presented in eTables 4, 5, and 6 in Supplement 1.

### Statistical Analysis

We assessed the balance of covariates across exposure groups using absolute standardized differences, with values less than 0.1 indicating acceptable balance.^22^ Propensity scores were estimated using logistic regression that included all prespecified covariates. Propensity score nearest neighbor 1 to 1 matching with a maximum caliper of 0.01 was applied and treatment effects were estimated without further adjustment. Cox proportional hazards regression was used to estimate unadjusted and adjusted hazard ratios (HRs). We used robust variance estimators to calculate 95% CIs. Incidence rates per 1000 person-years and adjusted HRs were calculated within each database and pooled across databases using fixed effects meta-analysis. Subgroup analyses were conducted based on 2 age strata: 6 to 11 years and 12 to 17 years. Additional subgroup analyses were performed within each IBD subtype.

Several sensitivity analyses were conducted to test the robustness of the primary findings. First, we repeated the analysis only among users of anti-TNF monotherapy, excluding patients who received immunomodulators (thiopurines or methotrexate) and oral corticosteroids within 180 days prior to cohort entry as well as censoring patients at the time they initiated any of these therapies during follow-up. Second, we excluded biosimilars for infliximab and adalimumab, limiting the analysis to patients treated with originator agents only. Third, we extended the follow-up period to 365 days. Fourth, we excluded gastrointestinal infections from the serious infection outcome given the potential for these diagnoses to be due to poorly controlled disease (eg, fistulizing CD resulting in an intra-abdominal abscess). Fifth, we restricted the analysis to patients with no observed use of corticosteroids, immunomodulators, aminosalicylates, or IBD-related biologics within 180 days before cohort entry (first-line users, distinct from the monotherapy sensitivity analysis by also excluding users of aminosalicylates). Sixth, we estimated high-dimensional propensity scores (HDPS), a data-driven approach to enhance confounding control by incorporating both predefined covariates and empirically selected variables. In addition to the investigator-prespecified covariates, the HDPS algorithm selected 150 empirical covariates from inpatient, outpatient, and pharmacy claims data during baseline. Candidate covariates were ranked using a bias-based algorithm and the resulting HDPS used for 1:1 nearest-neighbor matching.^23^ We evaluated tendinitis or tendinopathy as a negative control outcome because it was not expected to be associated with anti-TNF treatment choice and could help assess potential residual confounding related to differences in health care–seeking behavior.^24,25^

All analyses were prespecified and performed using Aetion Substantiate (2025) with R, version 4.3.3 (R Project for Statistical Computing), integration.^26,27^ We based interpretations of results on the magnitude of the point estimate and precision of the confidence interval for each analysis, rather than dichotomizing *P* values into significant and not significant, consistent with American Statistical Association guidance.^28^

## Results

### Study Population Characteristics

**Figure 1.**
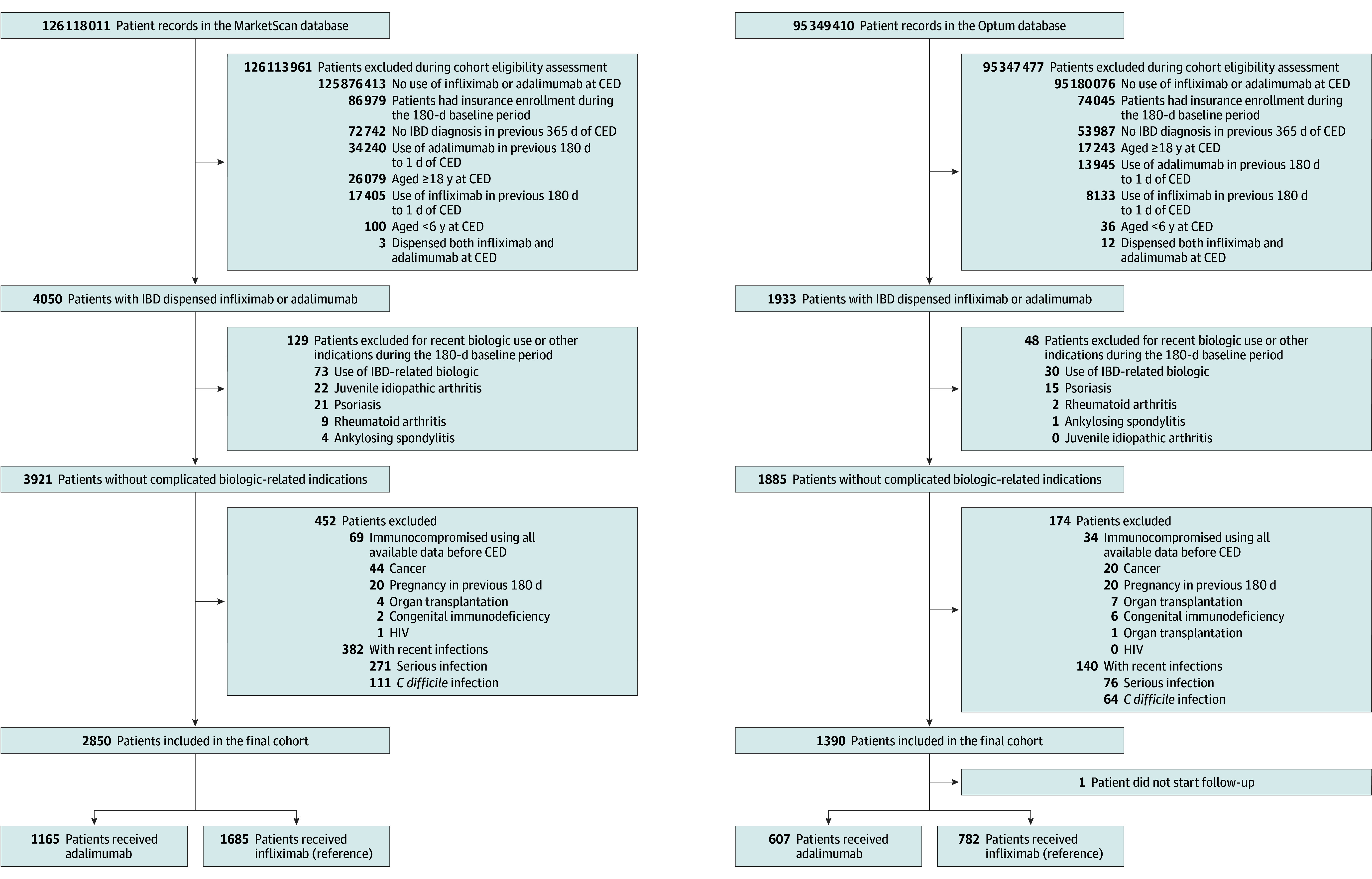
Flow Diagram of Patient Screening and Cohort Selection from Merative MarketScan Commercial Database and Optum Clinformatics Data Mart Database The flow diagram shows the identification of eligible patients from the Merative MarketScan Commercial Database and Optum Clinformatics Data Mart Database. Patients were screened based on exclusion criteria, including age, diagnosis codes, medication exposure, continuous enrollment, and recent biologic use or other indications. *C difficile* indicates *Clostridium difficile*; CED, cohort entry date; and IBD, inflammatory bowel disease.

There were 4050 children aged 6 to 17 years in MarketScan and 1933 in Optum who were new users of infliximab or adalimumab with an IBD diagnosis. After exclusions (Figure 1), we identified 2850 children initiating biologic therapy in MarketScan (1165 adalimumab and 1685 infliximab) and 1389 in Optum (607 adalimumab and 782 infliximab) (Table). A total of 2467 children (mean [SD] age, 13.3 [2.9] years; 1462 boys [59.3%] and 1005 girls [40.7%]; 1502 with CD [60.9%]) initiated infliximab and 1772 (mean [SD] age, 14.0 [2.7] years; 1047 boys [59.1%] and 725 girls [40.9%]; 1068 with CD [60.3%]) initiated adalimumab. Users of adalimumab were older, had fewer colonoscopies or sigmoidoscopies, were more likely to use baseline aminosalicylates, and had a lower prevalence of anemia, abnormal weight loss, and complicated IBD across databases.

**Table.** Baseline Clinical and Demographic Characteristics

Variable	MarketScan, No. (%)						Optum, No. (%)					
	Unmatched			Propensity score matched			Unmatched			Propensity score matched		
	Infliximab (n = 1685)	Adalimumab (n = 1165)	ASD	Infliximab (n = 1037)	Adalimumab (n = 1037)	ASD	Infliximab (n = 782)	Adalimumab (n = 607)	ASD	Infliximab (n = 496)	Adalimumab (n = 496)	ASD
**Demographic characteristics^a^**												
Age, y												
6-11	424 (25.2)	209 (17.9)	0.18	195 (18.8)	199 (19.2)	0.01	222 (28.4)	103 (17.0)	0.28	99 (20.0)	99 (20.0)	0.00
12-17	1261 (74.8)	956 (82.1)	0.18	842 (81.2)	838 (80.8)	0.01	560 (71.6)	504 (83.0)	0.28	397 (80.0)	397 (80.0)	0.00
Sex												
Female	681 (40.4)	489 (42.0)	0.03	432 (41.7)	432 (41.7)	0.00	324 (41.4)	236 (38.9)	0.05	197 (39.7)	201 (40.5)	0.02
Male	1004 (59.6)	676 (58.0)	0.03	605 (58.3)	605 (58.3)	0.00	458 (58.6)	371 (61.1)	0.05	299 (60.3)	295 (59.5)	0.02
**Clinical characteristics**												
IBD subtype												
Ulcerative colitis	337 (20.0)	273 (23.4)	0.08	222 (21.4)	230 (22.2)	0.02	154 (19.7)	139 (22.9)	0.08	107 (21.6)	106 (21.4)	0.01
Crohn disease	1023 (60.7)	691 (59.3)	0.03	633 (61.0)	620 (59.8)	0.03	479 (61.3)	377 (62.1)	0.02	307 (61.9)	306 (61.7)	0.01
Unspecified	325 (19.3)	201 (17.3)	0.05	182 (17.6)	187 (18.0)	0.01	149 (19.1)	91 (15.0)	0.11	82 (16.5)	84 (16.9)	0.01
IBD disease activity assessment												
Hospitalization for IBD^b^	148 (8.8)	49 (4.2)	0.19	49 (4.7)	47 (4.5)	0.01	34 (4.3)	14 (2.3)	0.11	14 (2.8)	13 (2.6)	0.01
Esophagoduodenoscopy	850 (50.4)	632 (54.2)	0.08	563 (54.3)	549 (52.9)	0.03	451 (57.7)	351 (57.8)	0.00	283 (57.1)	289 (58.3)	0.02
Colonoscopy or sigmoidoscopy	1270 (75.4)	857 (73.6)	0.04	772 (74.4)	760 (73.3)	0.03	614 (78.5)	452 (74.5)	0.10	373 (75.2)	374 (75.4)	0.01
CT of abdomen or pelvis	213 (12.6)	150 (12.9)	0.01	133 (12.8)	136 (13.1)	0.01	112 (14.3)	80 (13.2)	0.03	68 (13.7)	72 (14.5)	0.02
MR of abdomen or pelvis with contrast	718 (42.6)	395 (33.9)	0.18	387 (37.3)	372 (35.9)	0.03	356 (45.5)	230 (37.9)	0.16	196 (39.5)	199 (40.1)	0.01
C-reactive protein test ordered	1487 (88.2)	956 (82.1)	0.18	884 (85.2)	881 (85.0)	0.01	730 (93.4)	532 (87.6)	0.20	454 (91.5)	453 (91.3)	0.01
Gastrointestinal pathogen test ordered	196 (11.6)	139 (11.9)	0.01	130 (12.5)	120 (11.6)	0.03	149 (19.1)	94 (15.5)	0.10	82 (16.5)	85 (17.1)	0.02
**Baseline comorbidities**												
Pediatric Comorbidity Index, mean (SD)^c^	3.8 (2.5)	3.6 (2.4)	0.10	3.6 (2.4)	3.6 (2.4)	0.02	4.0 (2.5)	3.8 (2.4)	0.10	3.8 (2.5)	3.9 (2.4)	0.04
Anemia	584 (34.7)	317 (27.2)	0.16	295 (28.4)	300 (28.9)	0.01	317 (40.5)	182 (30.0)	0.22	164 (33.1)	166 (33.5)	0.01
Gastrointestinal bleeding	462 (27.4)	283 (24.3)	0.07	267 (25.7)	255 (24.6)	0.03	224 (28.6)	153 (25.2)	0.08	126 (25.4)	132 (26.6)	0.03
Abnormal weight loss	581 (34.5)	328 (28.2)	0.14	312 (30.1)	312 (30.1)	0.00	312 (39.9)	208 (34.3)	0.12	177 (35.7)	176 (35.5)	0.00
Protein-calorie malnutrition	114 (6.8)	46 (3.9)	0.13	52 (5.0)	43 (4.1)	0.04	65 (8.3)	27 (4.4)	0.16	25 (5.0)	26 (5.2)	0.01
Crohn disease with complications^d^	517 (30.7)	306 (26.3)	0.10	296 (28.5)	286 (27.6)	0.02	246 (31.5)	145 (23.9)	0.17	132 (26.6)	124 (25.0)	0.04
Ulcerative colitis with complications^d^	143 (8.5)	86 (7.4)	0.04	75 (7.2)	79 (7.6)	0.02	57 (7.3)	38 (6.3)	0.04	27 (5.4)	33 (6.7)	0.05
Underweight or failure to thrive	208 (12.3)	105 (9.0)	0.11	95 (9.2)	101 (9.7)	0.02	134 (17.1)	65 (10.7)	0.19	68 (13.7)	62 (12.5)	0.04
Obesity or overweight	41 (2.4)	38 (3.3)	0.05	33 (3.2)	27 (2.6)	0.04	22 (2.8)	17 (2.8)	0.00	13 (2.6)	15 (3.0)	0.02
**Medications**												
Previous use of thiopurines^e^	203 (12.0)	171 (14.7)	0.08	140 (13.5)	140 (13.5)	0	81 (10.4)	77 (12.7)	0.07	58 (11.7)	63 (12.7)	0.03
Concurrent use of thiopurines^f^	95 (5.6)	90 (7.7)	0.08	72 (6.9)	69 (6.7)	0.01	51 (6.5)	43 (7.1)	0.02	30 (6.0)	36 (7.3)	0.05
Previous use of methotrexate^e^	120 (7.1)	106 (9.1)	0.07	91 (8.8)	87 (8.4)	0.01	38 (4.9)	40 (6.6)	0.08	24 (4.8)	31 (6.3)	0.06
Concurrent use of methotrexate^f^	144 (8.5)	97 (8.3)	0.01	87 (8.4)	87 (8.4)	0.00	51 (6.5)	50 (8.2)	0.07	37 (7.5)	37 (7.5)	0.00
Previous use of aminosalicylates^e^	508 (30.1)	420 (36.1)	0.13	340 (32.8)	349 (33.7)	0.02	187 (23.9)	181 (29.8)	0.13	136 (27.4)	130 (26.2)	0.03
Concurrent use of aminosalicylates^f^	276 (16.4)	249 (21.4)	0.13	194 (18.7)	207 (20.0)	0.03	115 (14.7)	113 (18.6)	0.11	82 (16.5)	77 (15.5)	0.03
Systemic corticosteroids, mean (SD), mg equivalents^g^	599.4 (966.2)	610.6 (872.5)	0.01	610.3 (984.5)	607.1 (843.0)	0.00	544.5 (811.4)	521.9 (812.4)	0.03	534.2 (743.9)	544.6 (850.4)	0.01
Influenza vaccine	650 (38.6)	433 (37.2)	0.03	393 (37.9)	392 (37.8)	0.00	311 (39.8)	234 (38.6)	0.03	187 (37.7)	204 (41.1)	0.07
Extended pneumococcal vaccines	30 (1.8)	16 (1.4)	0.03	16 (1.5)	15 (1.4)	0.01	<11 (<1.4)	<11 (<1.8)	0.02	<11 (<2.2)	<11 (<2.2)	0.02
**Measures of health care use**												
No. of emergency department visits, mean (SD)	0.7 (1.6)	0.7 (1.4)	0.056	0.7 (1.5)	0.7 (1.4)	0.03	0.7 (1.6)	0.6 (1.2)	0.13	0.6 (1.3)	0.6 (1.2)	0.00
No. of outpatient visits, mean (SD)	5.6 (3.5)	5.4 (3.2)	0.055	5.4 (3.3)	5.5 (3.3)	0.01	5.6 (3.2)	5.4 (3.0)	0.06	5.4 (3.3)	5.6 (3.1)	0.05
No. of hospitalizations, mean (SD)^e^	0.1 (0.4)	0.1 (0.3)	0.196	0.1 (0.3)	0.1 (0.3)	0.02	0.1 (0.5)	0.1 (0.3)	0.14	0.1 (0.4)	0.1 (0.4)	0.01
Hospitalization days for IBD, mean (SD)^e^	0.7 (3.6)	0.2 (1.2)	0.179	0.2 (1.2)	0.2 (1.2)	0.00	0.9 (3.6)	0.4 (2.3)	0.15	0.6 (2.7)	0.5 (2.5)	0.02

Abbreviations: ASD, absolute standardized difference; CT, computed tomography; IBD, inflammatory bowel disease, MR, magnetic resonance.

^a^ Assessed on cohort entry.

^b^ Assessed from 180 days to 1 day prior to the date of cohort entry.

^c^ Assessed from 180 days prior to and including the date of cohort entry.

^d^ Crohn disease with complications and ulcerative colitis with complications were defined using *International Statistical Classification of Diseases and Related Health Problems, Tenth Revision*, diagnosis codes with complication subcategories (K50.01-K50.919 and K51.01-K51.919, restricted to complication codes listed in eTable 4 in Supplement 1).

^e^ Assessed from 180 days to 31 days prior to the date of cohort entry.

^f^ Assessed from 30 days to 1 day prior to the date of cohort entry.

^g^ Assessed from 60 days prior to the date of cohort entry.

After 1:1 propensity score matching there were 1533 patient pairs (1037 in MarketScan and 496 in Optum). All baseline characteristics were well balanced between groups (Table; eTables 7 and 8 in Supplement 1).

### Risk of Serious Infections

**Figure 2.**
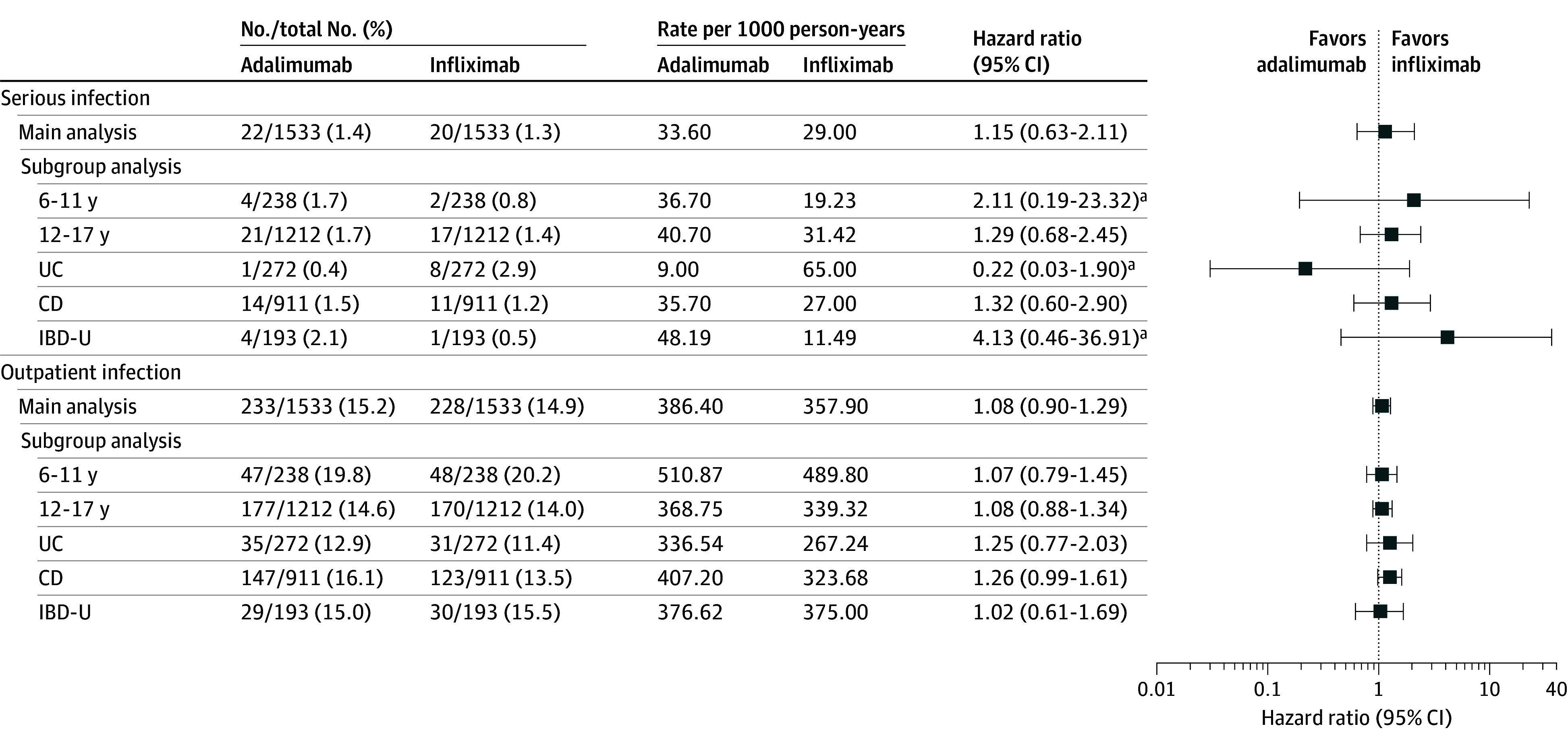
Forest Plot for Infection in the Main Analysis and Subgroup Analysis After Propensity Score Matching Forest plot showing pooled hazard ratios and 95% CIs for serious infections and outpatient infections comparing adalimumab with infliximab after propensity score matching in the MarketScan and Optum cohorts. Results are presented for the overall matched cohort and for subgroups defined by age group (6-11 years and 12-17 years) and inflammatory bowel disease subtype. Absolute event counts and incidence rates per 1000 person-years are also shown for each subgroup. Hazard ratios greater than 1 indicate a higher risk with adalimumab relative to infliximab. Error bars indicate 95% CIs. CD indicates Crohn disease; IBD-U, inflammatory bowel disease–unclassified; and UC, ulcerative colitis. ^a^From MarketScan database only, due to 0 events in Optum database.

After propensity score matching, 22 patients treated with adalimumab (incidence rate [IR], 34 per 1000 person-years) and 20 patients treated with infliximab (IR, 29 per 1000 person-years) had serious infections (pooled HR, 1.15; 95% CI, 0.63-2.11) (Figure 2). Skin and soft tissue infections were the most common infections, followed by serious gastrointestinal infections. No cases of meningitis were observed (eTable 9 in Supplement 1).

### Risk of Outpatient Infections

After propensity score matching, 233 patients treated with adalimumab (IR, 386 per 1000 person-years) and 228 patients treated with infliximab (IR, 358 per 1000 person-years) had outpatient infections requiring treatment (pooled HR, 1.08; 95% CI, 0.90-1.29) (Figure 2). Bacterial infections were the most frequent type of infection, followed by influenza (eTable 9 in Supplement 1). There were no mycobacterial infections identified in either group. Among outpatient bacterial infections, the most frequent were pharyngitis, acute sinusitis, skin and soft tissue infections, and acute otitis media (eTable 9 in Supplement 1).

### Subgroup Analyses

After age-stratified propensity score matching (Figure 2), there were 238 patients aged 6 to 11 years (children) per exposure group and 1212 patients aged 12 to 17 years (adolescents) per exposure group. Pooled HRs of serious infection were 2.11 (95% CI, 0.19-23.32) among children and 1.29 (95% CI, 0.68-2.45) among adolescents. Pooled HRs for outpatient infection were 1.07 (95% CI, 0.79-1.45) among children and 1.08 (95% CI, 0.88-1.34) among adolescents.

After propensity score matching within each IBD subtype, there were 272 patients per exposure group with UC, 911 patients per exposure group with CD, and 193 patients per exposure group with IBD-U (Figure 2). For serious infections, pooled HRs were 0.22 (95% CI, 0.03-1.90) among patients with UC, 1.32 (95% CI, 0.60-2.90) among patients with CD, and 4.13 (95% CI, 0.46-36.91) among patients with IBD-U. Serious infections occurred in 0.4% of adalimumab users with UC (1 of 272) vs 2.9% of infliximab users with UC (8 of 272), 1.5% of adalimumab users with CD (14 of 911) vs 1.2% of infliximab users with CD (11 of 911), and 2.1% of adalimumab users with IBD-U (4 of 193) vs 0.5% of infliximab users with IBD-U (1 of 193). For outpatient infections, pooled HRs were 1.25 (95% CI, 0.77-2.03) among patients with UC, 1.26 (95% CI, 0.99-1.61) among patients with CD, and 1.02 (95% CI, 0.61-1.69) in IBD-U.

### Sensitivity Analyses and Negative Control Outcomes Analyses

**Figure 3.**
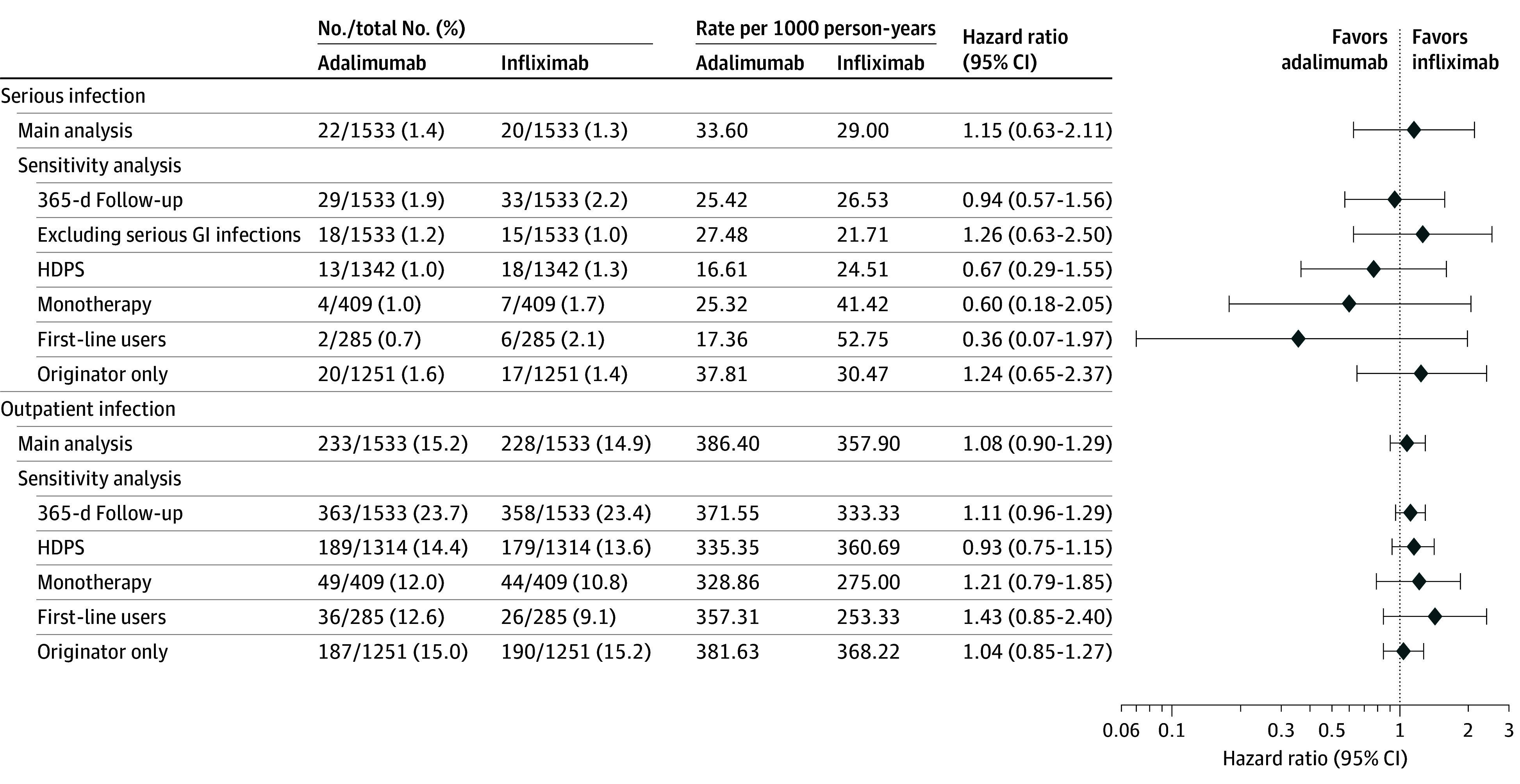
Forest Plots for Infections in the Main Analysis and Sensitivity Analysis After Propensity Score Matching Forest plot showing pooled hazard ratios and 95% CIs for serious infections and outpatient infections comparing adalimumab with infliximab after propensity score matching. Estimates are shown for the primary analysis and prespecified sensitivity analyses. Absolute event counts and incidence rates per 1000 person-years are also provided. Hazard ratios greater than 1 indicate a higher risk with adalimumab relative to infliximab. Error bars indicate 95% CIs. GI indicates gastrointestinal; and HDPS, high-dimensional propensity score matching analysis.

Overall, results of sensitivity analyses were consistent with those of primary analyses (Figure 3), with no difference in the risk of serious infections in the originator-only analysis (HR, 1.24; 95% CI, 0.65-2.37), with exclusion of serious gastrointestinal infections (HR, 1.26; 95% CI, 0.63-2.50), in the monotherapy-only analysis (HR, 0.60; 95% CI, 0.18-2.05), the extended follow-up period to 365 days (HR, 0.94; 95% CI, 0.57-1.56), in the first-line users analysis (HR, 0.36; 95% CI, 0.07-1.97), or in the HDPS matching analysis (HR, 0.67; 95% CI, 0.29-1.55). For outpatient infections, the primary analysis was not sensitive to restricting to monotherapy (HR, 1.21; 95% CI, 0.79-1.85), originator-only analysis (HR, 1.04; 95% CI, 0.85-1.27), 365 days of follow-up (HR, 1.11; 95% CI, 0.96-1.29), first-line users (HR, 1.43; 95% CI, 0.85-2.40), and HDPS matching (HR, 0.93; 95% CI, 0.75-1.15) (eTable 10 in Supplement 1). The control outcomes of tendinitis or tendinopathy showed no significant difference in risk between groups, with an unadjusted relative rate of 1.55 (95% CI, 0.72-3.34) and a propensity score–adjusted relative rate of 1.26 (95% CI, 0.55-2.91), suggesting improved balance between groups and no major differences in health care–seeking behavior (eTable 11 in Supplement 1).

## Discussion

This large, propensity score–matched, biologic-naive, active-comparator cohort study provides clinical evidence on the comparative safety of adalimumab vs infliximab as first biologic therapy for children and adolescents with IBD. Serious infections were rare, and the risk was similar with both agents, consistent with a systematic review of pediatric studies that reported a serious infection rate of 35.2 per 1000 person-years among children treated with anti-TNF agents.^29^ The low rate of serious infections aligns with RCTs in adults that compared each biologic with placebo.^30,31,32,33,34,35^

In open-label trials of infliximab vs placebo among children with IBD, serious infections ranged from 6.8% of patients with CD (7 of 103) to 13.3% of patients with UC (6 of 45),^34^ in contrast to adult placebo-controlled trials of infliximab that reported lower serious infection rates of 3.6% (14 of 385) among patients with CD and 2.1% (5 of 241) among patients with UC.^30,31^ In our study, serious infections occurred in 1.2% of infliximab users with CD (11 of 911) and 2.9% of patients with UC (8 of 272), consistent with the larger adult trials. In 2 pediatric RCTs comparing adalimumab against placebo, serious infection rates were 6.3% for patients with CD (12 of 192) and 5.4% for patients with UC (5 of 93).^5,6^ Similarly, adult RCTs reported lower serious infection rates than the pediatric studies, ranging from 0% (0 of 276) to 1.6% (4 of 257),^33,34^ more consistent with our findings of 1.5% (14 of 911) for patients with CD and 0.4% (1 of 272) for patients with UC. These comparisons should be interpreted cautiously as follow-up time, patient characteristics, and outcome definitions differed across studies. Inclusion of perirectal abscess as an infectious adverse event in pediatric trials may have increased the apparent frequency of serious infections, although perirectal abscesses may reflect underlying IBD activity rather than treatment-related infection. Overall, our findings may better reflect serious infection risk in routine clinical practice and do not suggest a markedly higher risk in children than that reported in adults.

Several prior observational studies in adults have compared the risk of serious infections between adalimumab and infliximab, yielding inconsistent results.^8,9,10^ Two studies used large US databases and found no difference in serious infection risk.^8,10^ A Danish cohort reported higher risk of serious infections with adalimumab in adult patients with UC (HR, 5.11; 95% CI, 1.20-21.80),^9^ although this risk was imprecisely estimated. Our study found no difference in serious infection risk between infliximab and adalimumab users, which persisted across subgroup and sensitivity analyses, reinforcing the robustness of this finding in the pediatric population and consistent with the 2 studies of adults conducted in the US.

We observed no difference in the risk of outpatient infections with adalimumab compared with infliximab (pooled HR, 1.08; 95% CI, 0.90-1.29). Direct comparisons with RCTs are limited, as most were placebo-controlled adult RCTs with narrow definitions of outpatient infections,^30,31,33,34^ and no head-to-head RCTs have been conducted in pediatric patients. In children with CD, adalimumab was associated with a modestly elevated point estimate for the risk of outpatient infections, but this was estimated imprecisely (HR, 1.26; 95% CI, 0.99-1.61). Across both treatment groups the most frequent outpatient infections were pharyngitis, acute sinusitis, and acute otitis media, similar to the distribution of outpatient infections most common in the general pediatric population.^36,37,38^

### Strengths and Limitations

This study has several strengths. To our knowledge, this is the first study to compare the risk of infections between adalimumab and infliximab among pediatric patients. Second, this is the largest cohort of either medication studied in children, enabling detection of rare serious infections. Third, we conducted subgroup and sensitivity analyses that confirmed the robustness of the primary analysis.

Despite these strengths, this study also has several limitations. First, residual confounding by unmeasured factors cannot be excluded, although HDPS used proxies of unmeasured factors to reduce confounding and was consistent with the primary analysis. Second, there is possible misclassification of outcomes, although serious infections were identified via an algorithm previously validated to have a positive predictive value of 80.2%, and outpatient infection diagnoses were paired with an antimicrobial prescription within 1 day to improve specificity. Third, despite the large cohort, we still have limited precision for estimates of serious infection subtypes given their infrequency. Fourth, while differences in health care–seeking behavior could affect the likelihood of seeking care for an outpatient infection, negative control analyses found no evidence of differential health care–seeking behavior between treatment groups. Fifth, this study was limited to commercially insured patients, which may limit generalizability to publicly insured populations, although we would not expect differential infection risk based on insurance status.

## Conclusions

In this cohort study of children and adolescents with IBD treated with adalimumab or infliximab, serious infections were rare while outpatient infections were more frequent. There was no evidence of a difference in serious or outpatient infection risk between groups, with findings consistent across several sensitivity analyses. These results suggest that infliximab and adalimumab may have comparable infection risk profiles for initial biologic treatment in pediatric IBD, although meaningful differences cannot be ruled out.

## Notes

Supplement 1. **eFigure. **Graphical Depiction of Study Design **eTable 1. **Exposure Definitions **eTable 2. **Exclusion Definitions **eTable 3. **Outcome Definitions **eTable 4. **Covariate Definitions **eTable 5. **Immunodeficiency Definition **eTable 6. **Pediatric Comorbidity Score **eTable 7. **Comprehensive Patient Characteristics, Merative MarketScan Commercial Database **eTable 8. **Comprehensive Patient Characteristics, Optum Clinformatics Data Mart Database **eTable 9. **Pooled Infection Component Risks Before and After Propensity Score Matching **eTable 10. **Empirically Selected Covariates Included in the High-Dimensional Propensity Score Model **eTable 11. **Pooled Negative Control Outcome Before and After Propensity Score Matching

Supplement 2.
Data Sharing Statement
