# Supplementary material for: Risk of Infections With Infliximab vs Adalimumab Among Children With Inflammatory Bowel Disease
Source: JAMA Netw Open. 2026 Jul 10;9(7):e2622684. doi: 10.1001/jamanetworkopen.2026.22684 (PMC13355144; doi:10.1001/jamanetworkopen.2026.22684)
Supplement: Supplement 1. — eFigure. Graphical Depiction of Study Design eTable 1. Exposure Definitions eTable 2. Exclusion Definitions eTable 3. Outcome Definitions eTable 4. Covariate Definitions eTable 5. Immunodeficiency Definition eTable 6. Pediatric Comorbidity Score eTable 7. Comprehensive Patient Characteristics, Merative MarketScan Commercial Database eTable 8. Comprehensive Patient Characteristics, Optum Clinformatics Data Mart Database eTable 9. Pooled Infection Component Risks Before and After Propensity Score Matching eTable 10. Empirically Selected Covariates Included in the High-Dimensional Propensity Score Model eTable 11. Pooled Negative Control Outcome Before and After Propensity Score Matching [file jamanetwopen-e2622684-s001.pdf]

Supplementary Online Content

Lyu N, Tracy M, Schneeweiss S, Savage TJ. Risk of infections with infliximab vs adalimumab among children with inflammatory bowel disease. *JAMA Netw Open.* 2026;9(7):e2622684. doi:10.1001/jamanetworkopen.2026.22684

eFigure. Graphical Depiction of Study Design
eTable 1. Exposure Definitions
eTable 2. Exclusion Definitions
eTable 3. Outcome Definitions
eTable 4. Covariate Definitions
eTable 5. Immunodeficiency Definition
eTable 6. Pediatric Comorbidity Score
eTable 7. Comprehensive Patient Characteristics, Merative MarketScan Commercial Database
eTable 8. Comprehensive Patient Characteristics, Optum Clinformatics Data Mart Database
eTable 9. Pooled Infection Component Risks Before and After Propensity Score Matching
eTable 10. Empirically Selected Covariates Included in the High-Dimensional Propensity Score Model
eTable 11. Pooled Negative Control Outcome Before and After Propensity Score Matching

This supplementary material has been provided by the authors to give readers additional information about their work.

eFigure. Graphical Depiction of Study Design

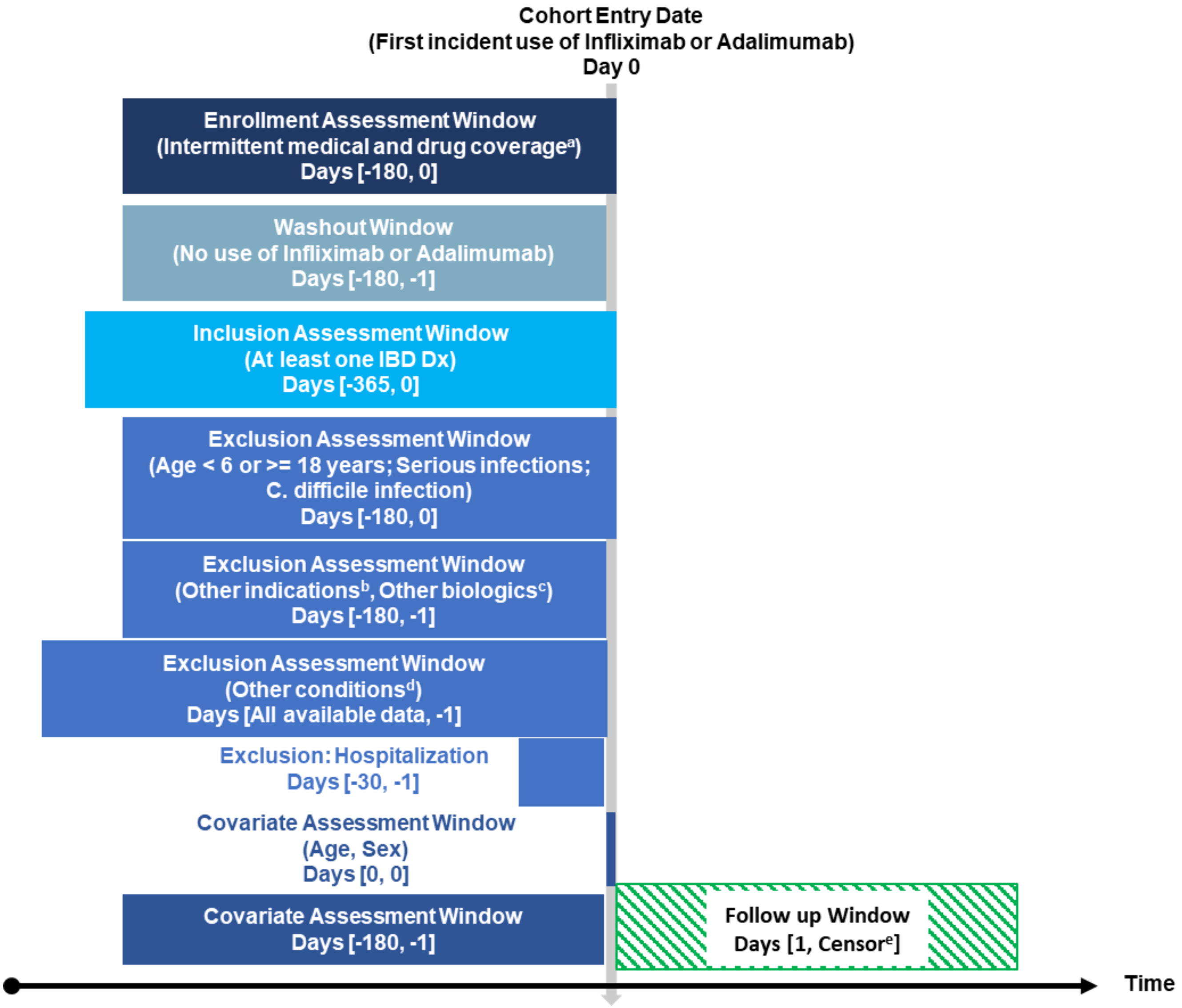

a. Up to 30-day gaps in medical or pharmacy enrollment allowed
b. Other indications included: Juvenile Rheumatoid Arthritis, rheumatoid arthritis, Psoriasis, ankylosing spondylitis, Psoriatic arthritis.
c. Other biologics indicated for IBD: Certolizumab pegol, Natalizumab, Vedolizumab, Ustekinumab, Risankizumab, Golimumab.
d. Other conditions: human immunodeficiency virus infection, congenital immunodeficiency, organ transplantation, history of cancer.
e. Earliest of outcome occurrence, death, plan disenrollment, end of study period, discontinuation of index exposure, or maximum 180 days follow-up period

© 2026 Lyu N et al. *JAMA Network Open.*

eTable 1. Exposure Definitions

Exposure	Drug Generic Name (Prescription Claims)	CPT/HCPCS Procedure Codes (Inpatient/outpatient)
Infliximab with biosimilars	INFLIXIMAB, INFLIXIMAB-ABDA, INFLIXIMAB-AXXQ, INFLIXIMAB-DYYB	J1745, Q5103, Q5104, Q5109, Q5121
Adalimumab with biosimilars	ADALIMUMAB, ADALIMUMAB-ATTO	J0135, J0139, Q5131, Q5132, Q5140, Q5141, Q5142, Q5143, Q5144, Q5145

© 2026 Lyu N et al. *JAMA Network Open.*

eTable 2. Exclusion Definitions

**Variable**	**Definition**[a]	**Assessment window** (relative to CED)
Inflammatory bowel disease	K50.00, K50.10, K50.80, K50.90, K51.00, K51.20, K51.30, K51.40, K51.50, K51.80, K51.90, K52.3	Day -365 to CED
IBD related Biologics[b,c]	USTEKINUMAB, VEDOLIZUMAB, CERTOLIZUMAB PEGOL, NATALIZUMAB, RISANKIZUMAB-RZAA, GOLIMUMAB<br><br>OR<br><br>C9261, J3357, J3358, 80280, C9026, J3380, C9249, J0717, J0718, C9126, J2323, Q4079, J2327, J1602	Day -180 to -1
Rheumatoid arthritis (RA)	M05 - M06	Day -180 to -1
Juvenile idiopathic arthritis (JIA)	M08	Day -180 to -1
Psoriatic arthritis	L40.51, L40.52, L40.53, L40.59	Day -180 to -1
Ankylosing spondylitis	M45	Day -180 to -1
Psoriasis	L40, L40.0, L40.1, L40.2, L40.3, L40.4, L40.8, L40.9	Day -180 to -1
Congenital immunodeficiency	D82.2, D82.3, D82.4, D82.8, D82.9	All available data to -1
Human immunodeficiency virus infection (HIV)	B20, B97.35, Z21	All available data to -1
Organ transplantation	02YA0Z0, 02YA0Z1, 02YA0Z2, 07YM0Z0, 07YM0Z1, 07YM0Z2, 07YP0Z0, 07YP0Z1, 07YP0Z2, 0BYC0Z0, 0BYC0Z1, 0BYC0Z2, 0BYD0Z0, 0BYD0Z1, 0BYD0Z2, 0BYF0Z0, 0BYF0Z1, 0BYF0Z2, 0BYG0Z0, 0BYG0Z1, 0BYG0Z2, 0BYH0Z0, 0BYH0Z1, 0BYH0Z2, 0BYJ0Z0, 0BYJ0Z1, 0BYJ0Z2, 0BYK0Z0, 0BYK0Z1, 0BYK0Z2, 0BYL0Z0, 0BYL0Z1, 0BYL0Z2, 0BYM0Z0, 0BYM0Z1, 0BYM0Z2, 0DY50Z0, 0DY50Z1, 0DY50Z2, 0DY60Z0, 0DY60Z1, 0DY60Z2, 0DY80Z0, 0DY80Z1, 0DY80Z2, 0DYE0Z0, 0DYE0Z1, 0DYE0Z2, 0FY00Z0, 0FY00Z1, 0FY00Z2, 0FYG0Z0, 0FYG0Z1, 0FYG0Z2, 0UY00Z0, 0UY00Z1, 0UY00Z2, 0UY10Z0, 0UY10Z1, 0UY10Z2, 0WY20Z0, 0WY20Z1, 0XYJ0Z0, 0XYJ0Z1, 0XYK0Z0, 0XYK0Z1, 3E030U0, 3E030U1, 3E033U0, 3E033U1, 3E0J3U0, 3E0J3U1, 3E0J7U0, 3E0J7U1, 3E0J8U0, 3E0J8U1	All available data to -1
Malignancy	C00, C01, C02, C03, C04, C05, C06, C07, C08, C09, C10, C11, C11.0, C12, C13, C14, C15, C16, C17, C18, C19, C20, C21, C22, C23, C24, C25, C26, C30, C31, C32, C33, C34, C37, C38, C39, C40, C41, C43, C45, C46, C47, C48, C49, C70, C71, C72, C73, C74, C75, C76, C77, C78, C79, C7A, C7B, C80, C81, C82, C83, C84, C85, C86, C88, C90, C91, C92, C93, C94, C95, C96, D03, D47.Z9, E31.21, E31.22, E31.23, R18.0	All available data to -1
Pregnancy[a,c]	O42.013, O43.121, O43.129, O43.231, O43.233, O64.1XX1, O64.2XX3, O64.2XX9, O64.3XX4, O64.4XX1, O64.5XX3, O64.8XX2, O64.9XX0, O64.9XX1, O64.9XX9, O65.2, O65.8, O65.9, O66.2, O66.41, O69.0XX2, O69.0XX3, O69.0XX5, O69.2XX0, O69.3XX3, O69.4XX0, O69.4XX5, O69.5XX1, O69.82X1, O69.82X3, O69.82X5, O69.82X9, O69.89X3, O69.9XX1, O69.9XX2, O69.9XX3, O70.0, O71.1, O71.9, O73.0, O73.1, O74.1, O74.6, O80, O89.5, P05.06, P05.07, P05.14, P07.16, Z39.0, O43.219, O62.2, O62.4, O64.0XX5, O64.1XX0, O64.1XX2, O64.3XX0, O64.3XX2, O64.3XX9, O64.4XX0, O64.4XX3, O64.4XX9, O64.5XX1, O64.5XX5, O64.8XX0, O64.8XX4, O65.1, O65.5, O66.40, O69.0XX4, O69.1XX1, O69.1XX3, O69.1XX4, O69.2XX5, O69.3XX1, O69.4XX2, O69.4XX3, O69.4XX9, O69.5XX2, O69.81X0, O69.81X5, O69.81X9, O69.82X2, O69.89X1, O69.89X5, O69.9XX0, O70.20, O70.21, O70.22, O71.02, O71.03, O71.2, O71.89, O74.7, O74.8, O74.9, O75.9, O89.01, O89.2, O89.8, P05.01, P05.03, P05.13, P05.16, P07.03, P07.1, P07.10, P07.14, P07.15, P07.17, P07.3, Z39.2, O42.01, O43.122, O43.213, O43.221, O43.222, O43.229, O43.232, O60.1, O62.0,	Day -180 to -1

© 2026 Lyu N et al. *JAMA Network Open.*

	O64.0XX9, O64.1XX4, O64.1XX5, O64.2XX0, O64.2XX4, O64.4XX4, O64.5XX4, O64.5XX9, O64.8XX1, O64.9XX2, O64.9XX4, O65.0, O65.3, O66.1, O66.5, O69.0XX0, O69.0XX9, O69.1XX0, O69.1XX9, O69.2XX4, O69.5XX0, O69.5XX3, O69.89X9, O69.9XX9, O70.3, O70.4, O70.9, O71.3, O71.4, O71.6, O74.3, O74.5, O89.4, P05.12, P05.15, P07.0, Z37.0, O42.019, O43.223, O62.8, O62.9, O63.2, O63.9, O64.0XX1, O64.0XX2, O64.0XX3, O64.2XX1, O64.2XX2, O64.2XX5, O64.3XX1, O64.3XX5, O64.4XX5, O64.8XX9, O66.0, O66.6, O66.8, O69.1XX5, O69.2XX1, O69.2XX3, O69.3XX2, O69.3XX5, O69.3XX9, O69.5XX9, O69.81X1, O69.81X4, O69.82X0, O69.82X4, O69.89X2, O69.89X4, O69.9XX5, O70.23, O71.5, O71.7, O71.81, O74.0, O74.2, O74.4, O89.09, O89.9, P05.04, P05.05, P05.11, P05.17, P07.01, P07.02, P61.2, H35.1, O43.123, O43.211, O43.212, O43.239, O62.1, O62.3, O63.0, O63.1, O64.0XX0, O64.0XX4, O64.1XX3, O64.1XX9, O64.3XX3, O64.4XX2, O64.5XX0, O64.5XX2, O64.8XX3, O64.8XX5, O64.9XX3, O64.9XX5, O65.4, O66.3, O66.9, O69.0XX1, O69.1XX2, O69.2XX2, O69.2XX9, O69.3XX0, O69.3XX4, O69.4XX1, O69.4XX4, O69.5XX4, O69.5XX5, O69.81X2, O69.81X3, O69.89X0, O69.9XX4, O70.1, O71.00, O71.82, O89.1, O89.3, O89.6, P05.02, P07.00, P07.30, Z39.1, O30, O30.0, O30.019, O30.043, O30.09, O30.099, O30.1, O30.10, O30.122, O30.192, O30.202, O30.211, O30.219, O30.231, O30.3003, O30.809, O30.812, O30.821, O30.93, O31.0, O31.01X3, O31.02X0, O31.10X0, O31.11X1, O31.11X9, O31.12X4, O31.12X5, O31.13X9, O31.20X2, O31.21X9, O31.23X9, O31.31, O31.31X3, O31.31X9, O31.32X3, O31.33, O31.33X1, O31.33X9, O31.8X2, O31.8X94, O43.02, P08.2, Z37.54, Z37.59, Z37.61, Z38.5, Z38.62, Z38.63, Z38.64, Z38.7, Z3A.41, O30.013, O30.02, O30.023, O30.032, O30.04, O30.093, O30.102, O30.111, O30.12, O30.139, O30.19, O30.199, O30.201, O30.222, O30.292, O30.332, O30.403, O30.803, O30.81, O30.813, O30.819, O30.82, O30.823, O30.831, O30.891, O30.91, O31.00X1, O31.00X2, O31.01X4, O31.01X5, O31.01X9, O31.02, O31.02X1, O31.02X3, O31.02X4, O31.02X9, O31.03X3, O31.03X4, O31.10X9, O31.11, O31.12X2, O31.12X3, O31.13X2, O31.13X5, O31.2, O31.20, O31.20X0, O31.20X3, O31.20X4, O31.20X9, O31.21, O31.21X, O31.23, O31.3, O31.31X5, O31.32X1, O31.32X2, O31.32X5, O31.421, O31.8X, O31.8X1, O31.8X10, O31.8X19, O31.8X92, O31.8X93, Z37.5, Z37.53, Z37.6, Z37.62, Z38.3, Z38.30, Z38.31, Z38.66, Z38.68, O30.00, O30.01, O30.011, O30.021, O30.022, O30.029, O30.041, O30.091, O30.092, O30.11, O30.113, O30.119, O30.121, O30.123, O30.191, O30.2, O30.20, O30.21, O30.221, O30.229, O30.232, O30.29, O30.291, O30.293, O30.80, O30.802, O30.811, O30.829, O30.89, O30.9, O31.00, O31.00X3, O31.01X0, O31.02X2, O31.02X5, O31.03, O31.03X0, O31.10X4, O31.11X3, O31.11X5, O31.12, O31.12X0, O31.12X1, O31.13, O31.13X0, O31.13X1, O31.13X4, O31.20X5, O31.21X0, O31.21X1, O31.21X2, O31.22, O31.22X1, O31.22X3, O31.22X4, O31.22X9, O31.23X0, O31.23X1, O31.23X3, O31.23X4, O31.23X5, O31.30X9, O31.31X0, O31.32X0, O31.32X4, O31.33X3, O31.33X4, O31.5, O31.8X13, O31.8X21, O31.8X29, O31.8X3, O31.8X32, O31.8X33, O31.8X34, O48.0, P01.5, Z37.52, Z37.64, Z37.69, Z38.6, Z38.69, Z3A.42, O30.001, O30.002, O30.003, O30.012, O30.03, O30.033, O30.042, O30.049, O30.112, O30.129, O30.133, O30.213, O30.22, O30.223, O30.8, O30.822, O30.892, O30.92, O30.981, O31.00X0, O31.01, O31.01X1, O31.03X5, O31.1, O31.10, O31.10X3, O31.10X5, O31.11X2, O31.11X4, O31.13X3, O31.21X3, O31.21X4, O31.21X5, O31.22X, O31.31X4, O31.32X9, O31.8, O31.8X11, O31.8X12, O31.8X15, O31.8X20, O31.8X25, O31.8X30, O31.8X31, O31.8X9, O31.8X99, O48.1, P08.22, Z37.2, Z37.3, Z37.50, Z37.60, Z38.4, Z38.61, Z38.65, Z38.8, O30.009, O30.031, O30.039, O30.101, O30.103, O30.109, O30.131, O30.132, O30.193, O30.203, O30.209, O30.212, O30.299, O30.41, O30.43, O30.801, O30.893, O30.899, O30.90, O31, O31.00X4, O31.00X5, O31.00X9, O31.01X2, O31.03X1, O31.03X2, O31.03X9, O31.10X1, O31.10X2, O31.11X0, O31.12X9, O31.20X1, O31.22X0, O31.22X2, O31.22X5, O31.23X2, O31.30, O31.30X0, O31.30X1, O31.30X2, O31.30X3, O31.30X4, O31.30X5, O31.31X1,	

© 2026 Lyu N et al. *JAMA Network Open.*

	O31.31X2, O31.32, O31.33X0, O31.33X2, O31.33X5, O31.8X14, O31.8X22, O31.8X23, O31.8X24, O31.8X35, O31.8X39, O31.8X90, O31.8X91, O31.8X95, O48, P08.21, Z37.51, Z37.63, Z3A.49, O09.0, O09.00, O09.01, O09.013, O09.02, O09.03, O09.0892, O09.1, O09.10, O09.11, O09.12, O09.13, O09.153, O09.2, O09.21, O09.211, O09.212, O09.213, O09.219, O09.2193, O09.22, O09.241, O09.29, O09.290, O09.291, O09.292, O09.293, O09.299, O09.3, O09.30, O09.31, O09.312, O09.32, O09.33, O09.4, O09.40, O09.41, O09.42, O09.43, O09.5, O09.500, O09.51, O09.511, O09.5113, O09.512, O09.5123, O09.513, O09.519, O09.52, O09.521, O09.521-, O09.522, O09.523, O09.529, O09.52X, O09.532, O09.54, O09.552, O09.573, O09.5921, O09.599, O09.5I2, O09.5X2, O09.6, O09.61, O09.611, O09.612, O09.613, O09.619, O09.62, O09.621, O09.622, O09.623, O09.629, O09.7, O09.70, O09.71, O09.72, O09.73, O09.8, O09.81, O09.811, O09.811-, O09.8111, O09.812, O09.813, O09.817, O09.819, O09.82, O09.821, O09.822, O09.823, O09.829, O09.83, O09.89, O09.891, O09.892, O09.893, O09.8933, O09.899, O09.89ED, O09.9, O09.90, O09.902, O09.91, O09.912, O09.92, O09.9212, O09.923, O09.93, O09.9619, O09.A0, O09.A1, O09.A2, O09.A3, O30.019, O30.043, O30.099, O30.122, O30.192, O30.202, O30.211, O30.219, O30.809, O30.812, O30.821, O30.93, O31.10X0, O31.11X1, O31.11X9, O31.12X4, O31.12X5, O31.13X9, O31.20X2, O31.21X9, O31.23X9, O31.31X3, O31.31X9, O31.32X3, O31.33X1, O31.33X9, O31.8X94, O32.9XX0, O32.9XX2, Z37.54, Z37.59, Z37.61, Z38.7, O30.013, O30.032, O30.093, O30.102, O30.111, O30.199, O30.201, O30.222, O30.292, O30.803, O30.813, O30.819, O30.823, O30.891, O30.91, O31.10X9, O31.12X2, O31.12X3, O31.13X2, O31.13X5, O31.20X0, O31.20X3, O31.20X4, O31.20X9, O31.31X5, O31.32X1, O31.32X2, O31.32X5, O31.8X10, O31.8X19, O31.8X92, O31.8X93, O32.9XX9, Z37.53, Z37.62, Z38.30, Z38.31, Z38.68, O30.011, O30.041, O30.091, O30.092, O30.113, O30.119, O30.121, O30.123, O30.191, O30.221, O30.229, O30.291, O30.293, O30.802, O30.811, O30.829, O31.10X4, O31.11X3, O31.11X5, O31.12X0, O31.12X1, O31.13X0, O31.13X1, O31.13X4, O31.20X5, O31.21X0, O31.21X1, O31.21X2, O31.22X1, O31.22X3, O31.22X4, O31.22X9, O31.23X0, O31.23X1, O31.23X3, O31.23X4, O31.23X5, O31.30X9, O31.31X0, O31.32X0, O31.32X4, O31.33X3, O31.33X4, O31.8X13, O31.8X21, O31.8X29, O31.8X32, O31.8X33, O31.8X34, P01.5, Z37.52, Z37.64, Z37.69, Z38.69, O30.001, O30.002, O30.003, O30.012, O30.033, O30.042, O30.049, O30.112, O30.129, O30.213, O30.223, O30.822, O30.892, O30.92, O31.10X3, O31.10X5, O31.11X2, O31.11X4, O31.13X3, O31.21X3, O31.21X4, O31.21X5, O31.31X4, O31.32X9, O31.8X11, O31.8X12, O31.8X15, O31.8X20, O31.8X25, O31.8X30, O31.8X31, O31.8X99, O66.6, Z37.2, Z37.3, Z37.50, Z37.60, Z38.4, Z38.61, O30.009, O30.031, O30.039, O30.101, O30.103, O30.109, O30.193, O30.203, O30.209, O30.212, O30.299, O30.801, O30.893, O30.899, O30.90, O31.10X1, O31.10X2, O31.11X0, O31.12X9, O31.20X1, O31.22X0, O31.22X2, O31.22X5, O31.23X2, O31.30X0, O31.30X1, O31.30X2, O31.30X3, O31.30X4, O31.30X5, O31.31X1, O31.31X2, O31.33X0, O31.33X2, O31.33X5, O31.8X14, O31.8X22, O31.8X23, O31.8X24, O31.8X35, O31.8X39, O31.8X90, O31.8X91, O31.8X95, O32.9XX1, O32.9XX3, O32.9XX4, O32.9XX5, Z37.51, Z37.63, O48.0, O48.1, P08.22, P08.21, O60.12X1, O60.13X0, O60.13X1, O60.13X2, O60.14X4, O60.14X5, P07.16, P07.25, P07.26, P07.32, P07.38, O60.12X5, O60.14X0, O60.14X1, O60.14X2, O60.22X0, O60.23X0, O60.23X1, O60.23X2, P07.03, P07.10, P07.14, P07.15, P07.17, P07.18, P07.22, P07.39, O60.12X0, O60.12X2, O60.13X3, O60.13X4, O60.13X5, O60.14X3, O60.22X1, P07.21, P07.34, P07.37, O60.12X4, O60.13X9, O60.22X2, O60.22X3, O60.22X4, O60.23X9, P07.01, P07.02, P07.23, P07.33, P07.35, O60.12X3, O60.12X9, O60.14X9, O60.22X5, O60.22X9, O60.23X3, O60.23X4, O60.23X5, P07.00, P07.24, P07.31, P07.36<br><br>Or	

© 2026 Lyu N et al. *JAMA Network Open.*

	01961, 49491, 49492, 59050, 59051, 59160, 59514, 59525, 59618, 59620, 00836, 01960, 01967, 01969, 59610, 99440, 58611, 59400, 59409, 59410, 59612, 59614, 59622, 01963, 59300, 59414, 59430, 59510, 59515, 99436, 01962, 01968, 58605, 59305, 59412, 67229, 99464	
Serious infection	A01.01, A02.21, A17.0, A20.3, A27.81, A32.11, A39.0, A42.81, A69.21, A87.0, A87.1, A87.2, A87.8, A87.9, B00.3, B01.0, B02.1, B05.1, B06.02, B26.1, B27.02, B27.12, B27.82, B27.92, B37.5, B38.4, B45.1, B57.41, G00.0, G00.1, G00.2, G00.3, G00.8, G00.9, G01, G02, G03.8, G03.9, A01.05, A02.24, H05.021, H05.022, H05.023, H05.029, M46.20, M46.21, M46.22, M46.23, M46.24, M46.25, M46.26, M46.27, M46.28, M86.00, M86.011, M86.012, M86.019, M86.021, M86.022, M86.029, M86.031, M86.032, M86.039, M86.041, M86.042, M86.049, M86.051, M86.052, M86.059, M86.061, M86.062, M86.069, M86.071, M86.072, M86.079, M86.08, M86.09, M86.10, M86.111, M86.112, M86.119, M86.121, M86.122, M86.129, M86.131, M86.132, M86.139, M86.141, M86.142, M86.149, M86.151, M86.152, M86.159, M86.161, M86.162, M86.169, M86.171, M86.172, M86.179, M86.18, M86.19, M86.20, M86.211, M86.212, M86.219, M86.221, M86.222, M86.229, M86.231, M86.232, M86.239, M86.241, M86.242, M86.249, M86.251, M86.252, M86.259, M86.261, M86.262, M86.269, M86.271, M86.272, M86.279, M86.28, M86.29, M86.8X0, M86.8X1, M86.8X2, M86.8X3, M86.8X4, M86.8X5, M86.8X6, M86.8X7, M86.8X8, M86.8X9, M86.9, A02.1, A32.7, A32.82, A39.2, A39.4, A39.51, A40.0, A40.1, A40.3, A40.8, A40.9, A41.01, A41.02, A41.1, A41.2, A41.3, A41.4, A41.50, A41.51, A41.52, A41.53, A41.59, A41.81, A41.89, A41.9, A42.7, A54.86, B33.21, B37.6, B37.7, I33.0, I76, M86.9, R65.20, R65.21, R78.81, A01.03, A02.22, A20.2, A21.2, A31.0, A37.01, A37.11, A37.81, A37.91, A43.0, A48.1, B01.2, B05.2, B06.81, B25.0, B37.1, B38.0, B38.2, B39.0, B39.2, B58.3, B59, B77.81, J10.00, J10.08, J11.00, J11.08, J12.0, J12.1, J12.2, J12.3, J12.81, J12.89, J12.9, J13, J14, J15.0, J15.1, J15.20, J15.211, J15.212, J15.29, J15.3, J15.4, J15.5, J15.6, J15.7, J15.8, J15.9, J16.0, J16.8, J17, J18.0, J18.1, J18.8, J18.9, J85.1, N10, N16 , A04.8, A04.9, A74.81, D73.3, K35.2, K35.3, K35.80, K35.89, K36, K37, K38.9, K55.30, K55.31, K55.32, K55.33, K57.00, K57.01, K57.12, K57.13, K57.20, K57.21, K57.32, K57.33, K57.40, K57.41, K57.52, K57.53, K57.80, K57.81, K57.92, K57.93, K63.0, K65.0, K65.1, K65.2, K65.3, K65.8, K65.9, K68.19, K75.0, K81.0, K81.2, K81.9, K85.0, K85.00, K85.01, K85.02, K85.1, K85.10, K85.11, K85.12, K85.3, K85.30, K85.31, K85.32, K85.8, K85.80, K85.81, K85.82, K85.9, K85.90, K85.91, K85.92, A31.1, A36.3, A43.1, A46, A48.0, H00.031, H00.032, H00.033, H00.034, H00.035, H00.036, H00.039, H05.011, H05.012, H05.013, H05.019, H60.00, H60.01, H60.02, H60.03, H60.10, H60.11, H60.12, H60.13, I70.261, I70.262, I70.263, I70.268, I70.269, I73.01, I96, J34.0, J85.0, K12.2, K61.0, K61.1, K61.2, K61.3, L01.00, L01.01, L01.02, L01.03, L01.09, L01.1, L02.01, L02.02, L02.03, L02.11, L02.12, L02.13, L02.211, L02.212, L02.213, L02.214, L02.215, L02.216, L02.219, L02.221, L02.222, L02.223, L02.224, L02.225, L02.226, L02.229, L02.231, L02.232, L02.233, L02.234, L02.235, L02.236, L02.239, L02.31, L02.32, L02.33, L02.411, L02.412, L02.413, L02.414, L02.415, L02.416, L02.419, L02.421, L02.422, L02.423, L02.424, L02.425, L02.426, L02.429, L02.431, L02.432, L02.433, L02.434, L02.435, L02.436, L02.439, L02.511, L02.512, L02.519, L02.521, L02.522, L02.529, L02.531, L02.532, L02.539, L02.611, L02.612, L02.619, L02.621, L02.622, L02.629, L02.631, L02.632, L02.639, L02.811, L02.818, L02.821, L02.828, L02.831, L02.838, L02.91, L02.92, L02.93, L03.011, L03.012, L03.019, L03.031, L03.032, L03.039, L03.111, L03.112, L03.113, L03.114, L03.115, L03.116, L03.119, L03.211, L03.213, L03.221, L03.311, L03.312, L03.313, L03.314, L03.315, L03.316, L03.317, L03.319, L03.811, L03.818, L03.90, L05.01, L05.02, L05.91, L05.92, L08.89, L08.9, M72.6, N48.21, N48.22, N61.1, N73.0, N73.2	Day -180 to CED
C.difficile infection	A04.72, A04.7	Day -180 to CED

© 2026 Lyu N et al. *JAMA Network Open.*

Any hospitalization	The occurrence of Inpatient	Day -30 to -1

[a]International Classification of Diseases – 10th Edition codes unless otherwise noted.
[b]Identified via generic names
[c]CPT codes

© 2026 Lyu N et al. *JAMA Network Open.*

eTable 3. Outcome Definitions

Serious infection outcome requiring hospitalization	
**Outcome component (any of following)**	**ICD-10 codes (Inpatient)**
Bacteremia	A02.1, A32.7, A32.82, A39.2, A39.4, A39.51, A40.0, A40.1, A40.3, A40.8, A40.9, A41.01, A41.02, A41.1, A41.2, A41.3, A41.4, A41.50, A41.51, A41.52, A41.53, A41.59, A41.81, A41.89, A41.9, A42.7, A54.86, B33.21, B37.6, B37.7, I33.0, I76, M86.9, R65.20, R65.21, R78.81
Pneumonia	A01.03, A02.22, A20.2, A21.2, A31.0, A37.01, A37.11, A37.81, A37.91, A43.0, A48.1, B01.2, B05.2, B06.81, B25.0, B37.1, B38.0, B38.2, B39.0, B39.2, B58.3, B59, B77.81, J10.00, J10.08, J11.00, J11.08, J12.0, J12.1, J12.2, J12.3, J12.81, J12.89, J12.9, J13, J14, J15.0, J15.1, J15.20, J15.211, J15.212, J15.29, J15.3, J15.4, J15.5, J15.6, J15.7, J15.8, J15.9, J16.0, J16.8, J17, J18.0, J18.1, J18.8, J18.9, J85.1
Skin/soft tissue infection	A31.1, A36.3, A43.1, A46, A48.0, H00.031, H00.032, H00.033, H00.034, H00.035, H00.036, H00.039, H05.011, H05.012, H05.013, H05.019, H60.00, H60.01, H60.02, H60.03, H60.10, H60.11, H60.12, H60.13, I70.261, I70.262, I70.263, I70.268, I70.269, I73.01, I96, J34.0, J85.0, K12.2, K61.0, K61.1, K61.2, K61.3, L01.00, L01.01, L01.02, L01.03, L01.09, L01.1, L02.01, L02.02, L02.03, L02.11, L02.12, L02.13, L02.211, L02.212, L02.213, L02.214, L02.215, L02.216, L02.219, L02.221, L02.222, L02.223, L02.224, L02.225, L02.226, L02.229, L02.231, L02.232, L02.233, L02.234, L02.235, L02.236, L02.239, L02.31, L02.32, L02.33, L02.411, L02.412, L02.413, L02.414, L02.415, L02.416, L02.419, L02.421, L02.422, L02.423, L02.424, L02.425, L02.426, L02.429, L02.431, L02.432, L02.433, L02.434, L02.435, L02.436, L02.439, L02.511, L02.512, L02.519, L02.521, L02.522, L02.529, L02.531, L02.532, L02.539, L02.611, L02.612, L02.619, L02.621, L02.622, L02.629, L02.631, L02.632, L02.639, L02.811, L02.818, L02.821, L02.828, L02.831, L02.838, L02.91, L02.92, L02.93, L03.011, L03.012, L03.019, L03.031, L03.032, L03.039, L03.111, L03.112, L03.113, L03.114, L03.115, L03.116, L03.119, L03.211, L03.213, L03.221, L03.311, L03.312, L03.313, L03.314, L03.315, L03.316, L03.317, L03.319, L03.811, L03.818, L03.90, L05.01, L05.02, L05.91, L05.92, L08.89, L08.9, M72.6, N48.21, N48.22, N61.1, N73.0, N73.2
Gastrointestinal infection	A04.8, A04.9, A74.81, D73.3, K35.2, K35.3, K35.80, K35.89, K36, K37, K38.9, K55.30, K55.31, K55.32, K55.33, K57.00, K57.01, K57.12, K57.13, K57.20, K57.21, K57.32, K57.33, K57.40, K57.41, K57.52, K57.53, K57.80, K57.81, K57.92, K57.93, K63.0, K65.0, K65.1, K65.2, K65.3, K65.8, K65.9, K68.19, K75.0, K81.0, K81.2, K81.9, K85.0, K85.00, K85.01, K85.02, K85.1, K85.10, K85.11, K85.12, K85.3, K85.30, K85.31, K85.32, K85.8, K85.80, K85.81, K85.82, K85.9, K85.90, K85.91, K85.92
Acute osteomyelitis	A01.05, A02.24, H05.021, H05.022, H05.023, H05.029, M46.20, M46.21, M46.22, M46.23, M46.24, M46.25, M46.26, M46.27, M46.28, M86.00, M86.011, M86.012, M86.019, M86.021, M86.022, M86.029, M86.031, M86.032, M86.039, M86.041, M86.042, M86.049, M86.051, M86.052, M86.059, M86.061, M86.062, M86.069, M86.071, M86.072, M86.079, M86.08, M86.09, M86.10, M86.111, M86.112, M86.119, M86.121, M86.122, M86.129, M86.131, M86.132, M86.139, M86.141, M86.142, M86.149, M86.151, M86.152, M86.159, M86.161, M86.162, M86.169, M86.171, M86.172, M86.179, M86.18, M86.19, M86.20, M86.211, M86.212, M86.219, M86.221, M86.222, M86.229, M86.231, M86.232, M86.239, M86.241, M86.242, M86.249, M86.251, M86.252, M86.259, M86.261, M86.262, M86.269, M86.271, M86.272, M86.279,

© 2026 Lyu N et al. *JAMA Network Open.*

	M86.28, M86.29, M86.8X0, M86.8X1, M86.8X2, M86.8X3, M86.8X4, M86.8X5, M86.8X6, M86.8X7, M86.8X8, M86.8X9, M86.9
Acute pyelonephritis	N10, N16
Acute meningitis	A01.01, A02.21, A17.0, A20.3, A27.81, A32.11, A39.0, A42.81, A69.21, A87.0, A87.1, A87.2, A87.8, A87.9, B00.3, B01.0, B02.1, B05.1, B06.02, B26.1, B27.02, B27.12, B27.82, B27.92, B37.5, B38.4, B45.1, B57.41, G00.0, G00.1, G00.2, G00.3, G00.8, G00.9, G01, G02, G03.8, G03.9
1. Outpatient infection requiring follow-up prescription	
**Outcome component (any of following)**	**ICD-10 codes (Outpatient) and Generic names**
Bacterial infections	H65.*, H66.*, J01.00, J01.10, J01.20, J01.30, J01.40, J01.80, J01.90, J20.*, J03.00, J03.01, A48.1, J13, J14, J15.*, J16.0, J16.8, J17, J18.0, J18.1, J18.2, J18.8, J18.9, L00, L01.*, L02.*, L03.*, L04.*, L05.*, L08.*, N10, N13.6, N30, N30.00, N30.01, N30.8, N30.80, N30.81, N30.9, N30.90, N30.91, N39.0<br><br>And<br><br>any of: {AMOXICILLIN, AMOXICILLIN/POTASSIUM CLAVULANATE, AZITHROMYCIN, AZITHROMYCIN HYDROGEN CITRATE, CEFACLOR, CEFADROXIL, CEFDINIR, CEFDITOREN PIVOXIL, CEFIXIME, CEFPODOXIME PROXETIL, CEFPROZIL, CEFTIBUTEN, CEFTRIAXONE SODIUM, CEFTRIAXONE SODIUM IN ISO-OSMOTIC DEXTROSE, CEFTRIAXONE SODIUM/LIDOCAINE HCL, CEFUROXIME AXETIL, CEPHALEXIN, CEPHALEXIN HCL, CIPROFLOXACIN, CIPROFLOXACIN HCL, CIPROFLOXACIN/CIPROFLOXACIN HCL, CLARITHROMYCIN, CLINDAMYCIN HCL, CLINDAMYCIN PALMITATE HCL, DELAFLOXACIN MEGLUMINE, DICLOXACILLIN SODIUM, DOXYCYCLINE CALCIUM, DOXYCYCLINE HYCLATE, DOXYCYCLINE MONOHYDRATE, ERYTHROMYCIN BASE, ERYTHROMYCIN ESTOLATE, ERYTHROMYCIN ETHYLSUCCINATE, ERYTHROMYCIN ETHYLSUCCINATE/SULFISOXAZOLE ACETYL, ERYTHROMYCIN STEARATE, LEVOFLOXACIN, LINEZOLID, MOXIFLOXACIN HCL, PENICILLIN V POTASSIUM, SULFAMETHOXAZOLE/TRIMETHOPRIM, TEDIZOLID PHOSPHATE, TELITHROMYCIN, TETRACYCLINE, TETRACYCLINE HCL}
Lyme	A69.2, A69.20, A69.21, A69.22, A69.23, A69.29<br><br>And<br><br>any of: {AMOXICILLIN, CEFTRIAXONE SODIUM, CEFTRIAXONE SODIUM IN ISO-OSMOTIC DEXTROSE, CEFTRIAXONE SODIUM/LIDOCAINE HCL, DOXYCYCLINE CALCIUM, DOXYCYCLINE HYCLATE, DOXYCYCLINE MONOHYDRATE}
Mycobacterial	A31.0, A31.1, A31.2, A31.8, A31.9<br><br>And<br><br>any of: {ISONIAZID, RIFAMPIN, RIFAPENTINE, RIFABUTIN, PYRAZINAMIDE}

© 2026 Lyu N et al. *JAMA Network Open.*

Yeast	B37, B37.0, B37.2, B37.3, B37.4, B37.41, B37.42, B37.5, B37.6, B37.7, B37.8, B37.81, B37.82, B37.83, B37.84, B37.9<br><br>And<br><br>any of: { CLOTRIMAZOLE, FLUCONAZOLE, KETOCONAZOLE, MICONAZOLE, NYSTATIN, OTESECONAZOLE}
Herpes	A60, A60.0, A60.00, A60.01, A60.02, A60.03, A60.04, A60.09, A60.1, A60.9, B00, B00.0, B00.1, B00.2, B00.3, B00.4, B00.5, B00.50, B00.51, B00.52, B00.53, B00.59, B00.7, B00.8, B00.81, B00.82, B00.89, B00.9<br><br>And<br><br>any of: {ACYCLOVIR, ACYCLOVIR SODIUM, ACYCLOVIR SODIUM IN 0.9 % SODIUM CHLORIDE, ACYCLOVIR SODIUM/DEXTROSE 5 % IN WATER, ACYCLOVIR, MICRONIZED, ACYCLOVIR/BENZYL ALCOHOL, ACYCLOVIR/HYDROCORTISONE, VALACYCLOVIR HCL, FAMCICLOVIR}
Influenza	J10, J10.0, J10.00, J10.01, J10.08, J10.1, J10.2, J10.8, J10.81, J10.82, J10.83, J10.89, J11, J11.0, J11.00, J11.08, J11.1, J11.2, J11.8, J11.81, J11.82, J11.83, J11.89<br><br>And<br><br>any of:{BALOXAVIR MARBOXIL, OSELTAMIVIR PHOSPHATE, PERAMIVIR/PF}
COVID	U07.1<br><br>And<br><br>any of:{MOLNUPIRAVIR, NIRMATRELVIR/RITONAVIR, REMDESIVIR}
2. Diagnostic codes for specific outpatient bacterial infections that are components of “Bacterial infections” above	
**Outcome component (any of following)**	**ICD-10 codes (Outpatient) and Antibiotics (code as bacterial infections)**
Acute otitis media	H65.0, H65.00, H65.01, H65.02, H65.03, H65.04, H65.05, H65.06, H65.07, H66.001, H66.002, H66.003, H66.004, H66.005, H66.006, H66.007, H66.009, H66.011, H66.012, H66.013, H66.014, H66.015, H66.016, H66.017, H66.019, H66.40, H66.41, H66.42, H66.43, H66.90, H66.91, H66.92, H66.93
Acute sinusitis	J01, J01.0, J01.00, J01.01, J01.1, J01.10, J01.11, J01.2, J01.20, J01.21, J01.3, J01.30, J01.31, J01.4, J01.40, J01.41, J01.8, J01.80, J01.81, J01.9, J01.90, J01.91
Acute bronchitis	J20, J20.0, J20.1, J20.2, J20.3, J20.4, J20.5, J20.6, J20.7, J20.8, J20.9, J40
Pharyngitis	J02, J02.0, J02.8, J02.9, J03.00, J03.01
Pneumonia	A48.1, J13, J14, J15.0, J15.1, J15.2, J15.20, J15.21, J15.211, J15.212, J15.29, J15.3, J15.4, J15.5, J15.6, J15.7, J15.8, J15.9, J16.0, J16.8, J17, J18.0, J18.1, J18.2, J18.8, J18.9
UTI	N10, N13.6, N30, N30.00, N30.01, N30.8, N30.80, N30.81, N30.9, N30.90, N30.91, N39.0
Skin and soft tissue infections	L00, L01, L01.0, L01.00, L01.01, L01.02, L01.03, L01.09, L01.1, L02, L02.0, L02.01, L02.02, L02.03, L02.1, L02.11, L02.12, L02.13, L02.2, L02.21, L02.211, L02.212, L02.213, L02.214,

	L02.215, L02.216, L02.219, L02.22, L02.221, L02.222, L02.223, L02.224, L02.225, L02.226, L02.229, L02.23, L02.231, L02.232, L02.233, L02.234, L02.235, L02.236, L02.239, L02.3, L02.31, L02.32, L02.33, L02.4, L02.41, L02.411, L02.412, L02.413, L02.414, L02.415, L02.416, L02.419, L02.42, L02.421, L02.422, L02.423, L02.424, L02.425, L02.426, L02.429, L02.43, L02.431, L02.432, L02.433, L02.434, L02.435, L02.436, L02.439, L02.5, L02.51, L02.511, L02.512, L02.519, L02.52, L02.521, L02.522, L02.529, L02.53, L02.531, L02.532, L02.539, L02.6, L02.61, L02.611, L02.612, L02.619, L02.62, L02.621, L02.622, L02.629, L02.63, L02.631, L02.632, L02.639, L02.8, L02.81, L02.811, L02.818, L02.82, L02.821, L02.828, L02.83, L02.831, L02.838, L02.9, L02.91, L02.92, L02.93, L03, L03.0, L03.01, L03.011, L03.012, L03.019, L03.02, L03.021, L03.022, L03.029, L03.03, L03.031, L03.032, L03.039, L03.04, L03.041, L03.042, L03.049, L03.1, L03.11, L03.111, L03.112, L03.113, L03.114, L03.115, L03.116, L03.119, L03.12, L03.121, L03.122, L03.123, L03.124, L03.125, L03.126, L03.129, L03.2, L03.21, L03.211, L03.212, L03.213, L03.22, L03.221, L03.222, L03.3, L03.31, L03.311, L03.312, L03.313, L03.314, L03.315, L03.316, L03.317, L03.319, L03.32, L03.321, L03.322, L03.323, L03.324, L03.325, L03.326, L03.327, L03.329, L03.8, L03.81, L03.811, L03.818, L03.89, L03.891, L03.898, L03.9, L03.90, L03.91, L04, L04.0, L04.1, L04.2, L04.3, L04.8, L04.9, L05, L05.0, L05.01, L05.02, L05.9, L05.91, L05.92, L08, L08.0, L08.1, L08.8, L08.81, L08.82, L08.89, L08.9

© 2026 Lyu N et al. *JAMA Network Open.*

eTable 4. Covariate Definitions

Covariate	ICD-10 codes	CPT codes	Medication Generic Names	Assessment window
**IBD disease activity assessment**				
Upper GI Endoscopy		00731, 00732, 00813, 0355T, 0651T, 3130F, 3132F, 43234, 43256, 43258, 44360, 44361, 44363, 44364, 44365, 44366, 44369, 44370, 44372, 44373, 44376, 44377, 44378, 44379, 91110, 91111, 91113		Day -180 to CED
Lower GI Endoscopy		00811, 00812, 00813, G9937, 45380, 45381, 45382, 45383, 45378, 0885T, 0886T, 45330, 45331, 45333, 45334, 45338, 45340, 45341, 45342, 45345, 45349		Day -180 to CED
CT abdomen or pelvis		72192, 72193, 72194, 74150, 74160, 74170, 74176, 74177, 74178		Day -180 to CED
MR Enterography		74183, 72197, 74182, 72195		Day -180 to CED
C-reactive protein tests ordered		86140, 86141, 0310U		Day -180 to CED
Gastrointestinal pathogen tests ordered		0369U, 87505, 87506, 87507		Day -365 to CED
**Baseline Comorbidities**				
Chronic kidney disease	N18, N18.1, N18.2, N18.3, N18.30, N18.31, N18.32, N18.4, N18.5, N18.9			All available data
Chronic liver disease	B00.81, B15, B15.0, B15.9, B16, B16.0, B16.1, B16.2, B16.9, B17, B17.0, B17.1, B17.10, B17.11, B17.2, B17.8, B17.9, B18, B18.0, B18.1, B18.2, B18.8, B18.9, B19, B19.0, B19.1, B19.10, B19.11, B19.2, B19.20, B19.21, B19.9, B25.1, I85.0,			All available data

© 2026 Lyu N et al. *JAMA Network Open.*

	I85.00, I85.01, I85.1, I85.10, I85.11, K71.3, K71.4, K71.5, K71.50, K71.51, K71.7, K72.10, K72.11, K72.9, K72.90, K72.91, K74.00, K74.01, K74.02, K74.2, K74.3, K74.4, K74.5, K74.6, K74.60, K74.69, K76.5, K76.7, K76.81, K76.82, K91.82, K91.83, P35.3, P58, P58.0, P58.1, P58.2, P58.3, P58.4, P58.41, P58.42, P58.5, P58.8, P58.9, P59, P59.0, P59.2, P59.20, P59.29, P59.3, P59.8, P59.9, P78.81, Q44.6, Q44.7, R17			
Asthma	J45.2, J45.20, J45.21, J45.22, J45.3, J45.30, J45.31, J45.32, J45.4, J45.40, J45.41, J45.42, J45.5, J45.50, J45.51, J45.52, J45.9, J45.90, J45.901, J45.902, J45.909, J45.99, J45.990, J45.991, J45.998			All available data
**IBD disease severity assessment**				
Anemia	D50, D50.0, D50.8, D50.9, D63.8, D64.9			Day -180 to CED
Gastrointestinal bleeding (Melena)	K92.1			Day -180 to CED
Abnormal weight loss	R63.4			Day -180 to CED
Protein-calorie Malnutrition	E43, E44, E44.0, E44.1, E46			Day -180 to CED
CD With Complications	K50.01, K50.018, K50.019, K50.11, K50.118, K50.119, K50.81, K50.818, K50.819, K50.91, K50.918, K50.919			Day -180 to CED
UC With Complications	K51.01, K51.018, K51.019, K51.21,			Day -180 to CED

© 2026 Lyu N et al. *JAMA Network Open.*

	K51.218, K51.219, K51.31, K51.318, K51.319, K51.41, K51.418, K51.419, K51.51, K51.518, K51.519, K51.81, K51.818, K51.819, K51.91, K51.918, K51.919			
Influenza vaccine			4037F, 4274F, 90630, 90653, 90654, 90655, 90656, 90657, 90658, 90659, 90660, 90661, 90662, 90663, 90664, 90666, 90667, 90668, 90672, 90673, 90674, 90682, 90685, 90686, 90687, 90688, 90689, 90694, 90756, G0008, G2163, G8108, G8482, G8636, G8639, G9141, G9142, Q2033, Q2034, Q2035, Q2036, Q2037, Q2038, Q2039	-365d to -1d
Extended pneumococcal vaccines			90732, 90677	-365d to -1d
Underweight/failure to thrive	R63.6, R62.51			Day -365 to CED
Obesity/overweight	E66.0, E66.01, E66.09, E66.1, E66.2, E66.8, E66.9, E66, E66.3			Day -365 to CED
**Medications (generic names unless otherwise noted)**				
Previous/ Concurrent Use of Thiopurines			AZATHIOPRINE, AZATHIOPRINE SODIUM, MERCAPTOPURINE, THIOGUANINE	Day-180 to -31/ Day-30 to CED
Previous/ Concurrent Use of Methotrexate		80204, J8610, J9250, J9260	METHOTREXATE, METHOTREXATE SODIUM, METHOTREXATE SODIUM/PF, METHOTREXATE/PF	Day-180 to -31/ Day-30 to CED
Previous/ Concurrent Use of Aminosalicylates			MESALAMINE, MESALAMINE WITH CLEANSING WIPES, SULFASALAZINE, OLSALAZINE SODIUM, BALSALAZIDE DISODIUM	Day-180 to -31/ Day-30 to CED
Systemic glucocorticoids daily dose, prednisone equivalent (mg)			Using following drug groups (dose conversion factors): PREDNISONE (1.0), TRIAMCINOLONE (1.25), METHYLPREDNISOLONE (1.25), HYDROCORTISONE (0.25), CORTISONE (0.25), DEXAMETHASONE (6.67), BETAMETHASONE (8.33), BUDESONIDE (4.55), PREDNISOLONE (1.0)	Day-60 to CED
Antibiotics			FIRST-LINE PENICILLIN: AMOXICILLIN, AMPICILLIN,	Day-180 to CED

© 2026 Lyu N et al. *JAMA Network Open.*

			DICLOXACILLIN, OXACILLIN, PENICILLIN V POTASSIUM<br>SECOND-LINE PENICILLIN: AMOXICILLIN-CLAVULANATE<br>FIRST-GENERATION MACROLIDE: ERYTHROMYCIN, ERYTHROMYCIN-SULFISOXAZOLE<br>SECOND-GENERATION MACROLIDE: AZITHROMYCIN, CLARITHROMYCIN, ROXITHROMYCIN<br>CEPHALOSPORIN: CEFACLOR, CEFADROXIL, CEFDINIR, CEFDITOREN, CEFIXIME,<br>CEFPODOXIME, CEFPROZIL, CEFTIBUTEN, CEFUROXIME, CEPHALEXIN, CEPHRADINE<br>TMP/SMX: TRIMETHOPRIM/SULFAMETHOXAZOLE<br>OTHER ANTIBIOTICS: CIPROFLOXACIN, CLINDAMYCIN, DOXYCYCLINE, GATIFLOXACIN, GEMIFLOXACIN, LEVOFLOXACIN, LINEZOLID, LOMEFLOXACIN, LORACARBEF, METRONIDAZOLE, MINOCYCLINE, MOXIFLOXACIN, NITROFURANTOIN, NORFLOXACIN, OFLOXACIN, SULFISOXAZOLE, TELITHROMYCIN, TETRACYCLINE, TRIMETHOPRIM, VANCOMYCIN	
Antivirals			ACYCLOVIR, ACYCLOVIR SODIUM, ACYCLOVIR SODIUM IN 0.9 % SODIUM CHLORIDE,<br>ACYCLOVIR SODIUM/DEXTROSE 5 % IN WATER, ACYCLOVIR, MICRONIZED, ACYCLOVIR/BENZYL ALCOHOL, ACYCLOVIR/HYDROCORTISONE, VALACYCLOVIR<br>HCL, FAMCICLOVIR	
Antifungals		80187, 80189, 80285, J0285, J0286, J0287, J0288, J0289, J1450, J1833, J1835, J3465, S0029, S0096	CLOTRIMAZOLE, FLUCONAZOLE, FLUCONAZOLE IN DEXTROSE, ISO-OSMOTIC,<br>FLUCONAZOLE IN SODIUM CHLORIDE, ISO-OSMOTIC,	

© 2026 Lyu N et al. *JAMA Network Open.*

			ISAVUCONAZONIUM SULFATE, ITRACONAZOLE, ITRACONAZOLE, MICRONIZED, KETOCONAZOLE, KETOCONAZOLE, MICRONIZED, MICONAZOLE, OTESECONAZOLE, POSACONAZOLE, VORICONAZOLE, MICAFUNGIN SODIUM, CASPOFUNGIN ACETATE, ANIDULAFUNGIN, TERBINAFINE, TERBINAFINE HCL, GRISEOFULVIN, GRISEOFULVIN ULTRAMICROSIZE, GRISEOFULVIN, MICROSIZE, IBREXAFUNGERP CITRATE, FLUCYTOSINE, NYSTATIN, AMPHOTERICIN B, AMPHOTERICIN B CHOLESTERYL SULFATE, AMPHOTERICIN B LIPID COMPLEX, AMPHOTERICIN B LIPOSOME	
**Healthcare utilization**				
No. of outpatient visits			Outpatient claim with CPT codes: 99201, 99202, 99203, 99204, 99205, 99211, 99212, 99213, 99214, 99215, 99241, 99242, 99243, 99244, 99245, 99385, 99386, 99387, 99395, 99396, 99397	-180d to -1d
No. of ED visits	Any of the following:<br>Outpatient procedure group: 111<br>Outpatient revenue code: 0450, 0451, 0452, 0456, 0459, 0981<br>Outpatient place of service: Emergency Room – Hospital<br>Outpatient – Service sub-category code: 10120, 10220,10320, 10420, 10520, 12220, 20120, 20220, 21120, 21220, 22120, 22320, 30120, 30220, 30320, 30420, 30520, 30620, 31120, 31220, 31320, 31420, 31520, 31620<br>Inpatient CPT code: 99281, 99282, 99283, 99284, 99285, 99288<br>Outpatient CPT code: 99281, 99282, 99283, 99284, 99285, 99288			-180d to -1d
No. of hospitalization			Inpatient claim	-180d to -1d

Definitions require subjects to have one or more codes or medications to be assigned as having the covariate. ICD-10 codes listed include all nest codes unless otherwise noted.
[a]Requires two or more codes

© 2026 Lyu N et al. *JAMA Network Open.*

eTable 5. Immunodeficiency Definition

**Immunodeficiency definition (any of the following)**			
	ICD-10	Medications	
HIV/AIDS	B20, Z21	ENFUVIRTIDE, ETRAVIRINE, LOPINAVIR/RITONAVIR, MARAVIROC, NEVIRAPINE, RILPIVIRINE, RITONAVIR, SAQUINAVIR, TIPRANAVIR, ZIDOVUDINE, ABACAVIR SULFATE, ATAZANAVIR SULFATE, COBICISTAT, ATAZANAVIR SULFATE/COBICISTAT, DARUNAVIR ETHANOLATE, DARUNAVIR ETHANOLATE/COBICISTAT, EFAVIRENZ, EMTRICITABINE, FOSAMPRENAVIR CALCIUM, INDINAVIR SULFATE, LAMIVUDINE, LAMIVUDINE/ZIDOVUDINE, ABACAVIR SULFATE/DOLUTEGRAVIR SODIUM/LAMIVUDINE, ABACAVIR SULFATE/LAMIVUDINE, ABACAVIR SULFATE/LAMIVUDINE/ZIDOVUDINE, DOLUTEGRAVIR SODIUM/LAMIVUDINE, NELFINAVIR MESYLATE, RALTEGRAVIR POTASSIUM, TENOFOVIR ALAFENAMIDE, TENOFOVIR DISOPROXIL FUMARATE, BICTEGRAVIR SODIUM/EMTRICITABINE/TENOFOVIR ALAFENAMIDE FUMAR, DARUNAVIR ETH/COBICISTAT/EMTRICITABINE/TENOFOVIR ALAFENAMIDE, DORAVIRINE/LAMIVUDINE/TENOFOVIR DISOPROXIL FUMARATE, EFAVIRENZ/EMTRICITABINE/TENOFOVIR DISOPROXIL FUMARATE, EFAVIRENZ/LAMIVUDINE/TENOFOVIR DISOPROXIL FUMARATE, ELVITEGRAVIR/COBICISTAT/EMTRICITABINE/TENOFOVIR ALAFENAMIDE, ELVITEGRAVIR/COBICISTAT/EMTRICITABINE/TENOFOVIR DISOPROXIL, EMTRICITABINE/RILPIVIRINE HCL/TENOFOVIR ALAFENAMIDE FUMARATE, EMTRICITABINE/RILPIVIRINE HCL/TENOFOVIR DISOPROXIL FUMARATE, EMTRICITABINE/TENOFOVIR ALAFENAMIDE FUMARATE, EMTRICITABINE/TENOFOVIR DISOPROXIL FUMARATE, LAMIVUDINE/TENOFOVIR DISOPROXIL FUMARATE	All available data to CED
	Medications		
Malignancy or myelodysplastic syndromes	aflibercept, aldesleukin, arsenic trioxide, asparaginase, atezolizumab, avelumab, azacitidine, belinostat, bendamustine, bevacizumab, bleomycin, blinatumomab, bortezomib, brentuximab, cabazitaxel, calaspargase, carboplatin, carfilzomib, carmustine, cemiplimab, cetuximab, cisplatin, cladribine, clofarabine, copanlisib, cyclophosphamide, cytarabine, dacarbazine, dactinomycin, daratumumab, daunorubicin, degarelix, denileukin, docetaxel, doxorubicin, durvalumab, elliott, elotuzumab, emapalumab, enfortumab, epirubicin, eribulin, etoposide, floxuridine, fludarabine, fluorouracil, fulvestrant, gemcitabine, gemtuzumab ozogamicin, goserelin, histrelin, hyaluronidase, idarubicin, ifosfamide, inotuzumab ozogamicin, ipilimumab, irinotecan, ixabepilone, leuprolide, mechlorethamine, melphalan, mesna, mitomycin, mitoxantrone, mogamulizumab, moxetumomab, necitumumab, nelarabine, nivolumab, obinutuzumab, ofatumumab, olaratumab, omacetaxine mepesuccinate, oxaliplatin, paclitaxel, panitumumab, pegaspargase, pembrolizumab, pemetrexed, pentostatin, pertuzumab, polatuzumab, porfimer, pralatrexate, ramucirumab, rituximab, romidepsin, streptozocin, tagraxofusp, temozolomide, temsirolimus, thiotepa, topotecan, trabectedin, trastuzumab, valrubicin, vinblastine, vincristine, vinorelbine		-180d to CED
	ICD-10	CPT Codes	
Hematologic / solid organ malignancies	C00.0-C43.9, C45.0-C49.0, C70-C79, C7A, C7B, C80-C96, D03,	J9000, J9015, J9017, J9019, J9020, J9022, J9023, J9025, J9027, J9032, J9033, J9034, J9035, J9036, J9039, J9040, J9041, J9042, J9043, J9044, J9045, J9047, J9050, J9055, J9057, J9060, J9065, J9070, J9098, J9100, J9118, J9119, J9120, J9130, J9145, J9150, J9151, J9153, J9155, J9160, J9171, J9173, J9175, J9176, J9177, J9178, J9179, J9181, J9185, J9190, J9198, J9200,	-365d to CED

© 2026 Lyu N et al. *JAMA Network Open.*

	D47.Z9, E31.21-E31.23, R18.0	J9201, J9202, J9203, J9204, J9205, J9206, J9207, J9208, J9209, J9210, J9211, J9217, J9218, J9219, J9225, J9226, J9228, J9229, J9230, J9245, J9246, J9261, J9262, J9263, J9264, J9266, J9267, J9268, J9269, J9271, J9280, J9285, J9293, J9295, J9299, J9301, J9302, J9303, J9305, J9306, J9307, J9308, J9309, J9311, J9312, J9313, J9315, J9320, J9328, J9330, J9340, J9351, J9352, J9354, J9355, J9356, J9357, J9358, J9360, J9370, J9371, J9390, J9395, J9400, J9600	
Aplastic anemias	ICD-10		
	D60.0-D61.9, D70.0, D71		All available data to CED
Hereditary immunodeficiency	ICD-10		
	D80-D85, D89-D89.49, D89.89, D89.90, D72.0		All available data to CED
Hemophagocytic Syndromes	ICD-10		
	D76.1-D76.3		All available data to CED
Sarcoidosis	ICD-10		
	D86		All available data to CED
Polyarteritis nodosa and related conditions	ICD-10		
	M30.0, M31.0-M31.1, M31.30-M31.31, M31.4, M31.6		All available data to CED
Diffuse diseases of connective tissue	ICD-10		
	M34.0-M34.1, M34.9		All available data to CED
Transplantation	ICD-10		
	Z48.21-Z48.298, Z94.0-Z94.9		All available data to CED

Abbreviations: CPT, Current Procedural Terminology; HIV/AIDS, human immunodeficiency virus/acquired immunodeficiency syndrome.

© 2026 Lyu N et al. *JAMA Network Open.*

eTable 6. Pediatric Comorbidity Score

Component	Weight
Alcohol abuse	1
Anemia	2
Anxiety	1
Any malignancy	5
Asthma	1
Chromosomal abnormalities	2
Cardiovascular abnormalities	2
Conduct disorder	1
Congenital malformations	2
Depression	4
Developmental delays	1
Diabetes	4
Drug abuse	3
Eating disorder	1
Epilepsy or convulsions	4
Gastrointestinal conditions	1
Joint disorders	1
Menstrual disorders	2
Nausea vomiting	1
Pain Conditions	1
Psychotic disorders	3
Sleep disorder	1
Smoking	2
Weight loss	2

© 2026 Lyu N et al. *JAMA Network Open.*

eTable 7. Comprehensive Patient Characteristics, Merative MarketScan Commercial Database

	Unmatched			Propensity-score Matched		
**Variable**	**Adalimumab (n=1,165)[a]**	**Infliximab (n= 1,685)[a]**	**ASD[b]**	**Adalimumab (n=1,037)[a]**	**Infliximab (n=1,037)[a]**	**ASD[b]**
**Demographic characteristics[c]**						
Year of Cohort Entry Date						
2016	89 (7.6)	152 (9.0)	0.05	88 (8.5)	79 (7.6)	0.03
2017	147 (12.6)	307 (18.2)	0.16	143 (13.8)	144 (13.9)	0.00
2018	168 (14.4)	274 (16.3)	0.05	150 (14.5)	147 (14.2)	0.01
2019	183 (15.7)	224 (13.3)	0.07	158 (15.2)	159 (15.3)	0.00
2020	162 (13.9)	248 (14.7)	0.02	148 (14.3)	152 (14.7)	0.01
2021	166 (14.2)	200 (11.9)	0.07	142 (13.7)	144 (13.9)	0.01
2022	175 (15.0)	183 (10.9)	0.12	141 (13.6)	140 (13.5)	0.00
2023	75 (6.4)	97 (5.8)	0.03	67 (6.5)	72 (6.9)	0.02
Age; mean (SD)	14.02 (2.7)	13.42 (2.9)	0.22	13.93 (2.7)	13.76 (2.7)	0.06
Age						
6 - 11	209 (17.9)	424 (25.2)	0.18	199 (19.2)	195 (18.8)	0.01
12 - 17	956 (82.1)	1,261 (74.8)	0.18	838 (80.8)	842 (81.2)	0.01
Sex						
Male	676 (58.0)	1,004 (59.6)	0.03	605 (58.3)	605 (58.3)	0.00
Female	489 (42.0)	681 (40.4)	0.03	432 (41.7)	432 (41.7)	0.00
Region						
South	532 (45.7)	613 (36.4)	0.19	452 (43.6)	447 (43.1)	0.01
North Central	265 (22.7)	414 (24.6)	0.04	242 (23.3)	243 (23.4)	0.00
Northeast	210 (18.0)	393 (23.3)	0.13	197 (19.0)	197 (19.0)	0.00
West	152 (13.0)	251 (14.9)	0.05	140 (13.5)	143 (13.8)	0.01
Others	6 (0.5)	14 (0.8)	0.04	6 (0.6)	7 (0.7)	0.01
Insurance type						
HMO	131 (11.2)	191 (11.3)	0.00	118 (11.4)	123 (11.9)	0.02
POS	91 (7.8)	158 (9.4)	0.06	85 (8.2)	71 (6.8)	0.05

© 2026 Lyu N et al. *JAMA Network Open.*

PPO	570 (48.9)	781 (46.4)	0.05	502 (48.4)	499 (48.1)	0.01
Others	345 (29.6)	521 (30.9)	0.03	305 (29.4)	319 (30.8)	0.03
Unknown	28 (2.4)	34 (2.0)	0.03	27 (2.6)	25 (2.4)	0.01
**Clinical characteristics**[d]						
IBD subtype						
Crohn's disease	691 (59.3)	1,023 (60.7)	0.03	620 (59.8)	633 (61.0)	0.03
Ulcerative colitis	273 (23.4)	337 (20.0)	0.08	230 (22.2)	222 (21.4)	0.02
Unspecified	201 (17.3)	325 (19.3)	0.05	187 (18.0)	182 (17.6)	0.01
IBD disease activity assessment						
C-reactive protein tests ordered	956 (82.1)	1,487 (88.2)	0.18	881 (85.0)	884 (85.2)	0.01
Colonoscopy/sigmoidoscopy	857 (73.6)	1,270 (75.4)	0.04	760 (73.3)	772 (74.4)	0.03
Esophagoduodenoscopy	632 (54.2)	850 (50.4)	0.08	549 (52.9)	563 (54.3)	0.03
MR abdomen or pelvis with contrast	395 (33.9)	718 (42.6)	0.18	372 (35.9)	387 (37.3)	0.03
CT abdomen or pelvis	150 (12.9)	213 (12.6)	0.01	136 (13.1)	133 (12.8)	0.01
Gastrointestinal pathogen tests ordered	139 (11.9)	196 (11.6)	0.01	120 (11.6)	130 (12.5)	0.03
Hospitalization for IBD[e]	49 (4.2)	148 (8.8)	0.19	47 (4.5)	49 (4.7)	0.01
**Baseline Comorbidities**[d]						
Pediatric comorbidity index; mean (SD)	3.58 (2.39)	3.82 (2.49)	0.10	3.57 (2.37)	3.61 (2.42)	0.02
CD With Complications	306 (26.3)	517 (30.7)	0.10	286 (27.6)	296 (28.5)	0.02
UC With Complications	86 (7.4)	143 (8.5)	0.04	79 (7.6)	75 (7.2)	0.02
Abnormal weight loss	328 (28.2)	581 (34.5)	0.14	312 (30.1)	312 (30.1)	0.00
Anemia	317 (27.2)	584 (34.7)	0.16	300 (28.9)	295 (28.4)	0.01
Gastrointestinal bleeding	283 (24.3)	462 (27.4)	0.07	255 (24.6)	267 (25.7)	0.03
Asthma	191 (16.4)	280 (16.6)	0.01	164 (15.9)	179 (17.4)	0.01
Underweight/failure to thrive	105 (9.0)	208 (12.3)	0.11	101 (9.7)	95 (9.2)	0.02
Immunodeficiency	62 (5.3)	115 (6.8)	0.06	58 (5.6)	54 (5.2)	0.02
Protein-calorie Malnutrition	46 (3.9)	114 (6.8)	0.13	43 (4.1)	52 (5.0)	0.04
Obesity/overweight	38 (3.3)	41 (2.4)	0.05	27 (2.6)	33 (3.2)	0.04
Chronic liver disease	5 (0.4)	14 (0.8)	0.05	5 (0.5)	4 (0.4)	0.02
Chronic kidney disease	2 (0.2)	4 (0.2)	0.02	2 (0.2)	2 (0.2)	0.00
**Baseline medications**						
Aminosalicylates, previous use[e]	420 (36.1)	508 (30.1)	0.13	349 (33.7)	340 (32.8)	0.02
Aminosalicylates, concurrent use[f]	249 (21.4)	276 (16.4)	0.13	207 (20.0)	194 (18.7)	0.03

© 2026 Lyu N et al. *JAMA Network Open.*

Thiopurines, previous use[e]	171 (14.7)	203 (12.0)	0.08	140 (13.5)	140 (13.5)	0.00
Thiopurines, concurrent use[f]	90 (7.7)	95 (5.6)	0.08	69 (6.7)	72 (6.9)	0.01
Methotrexate, previous use[e]	106 (9.1)	120 (7.1)	0.07	87 (8.4)	91 (8.8)	0.01
Methotrexate, concurrent use[f]	97 (8.3)	144 (8.5)	0.01	87 (8.4)	87 (8.4)	0.00
Systemic glucocorticosteroids; mean (SD)[g,h]	610.57 (872.50)	599.37 (966.21)	0.01	607.10 (843.02)	610.25 (984.53)	0.00
Days of Antibiotics; mean (SD)[d]	8.25 (19.60)	8.70 (18.24)	0.02	8.38 (20.28)	8.32 (17.46)	0.00
Days of Antivirals; mean (SD)[d]	0.11 (2.63)	0.15 (2.83)	0.01	0.12 (2.79)	0.05 (0.71)	0.04
Days of antifungals; mean (SD)[d]	0.80 (6.60)	0.90 (7.07)	0.01	0.76 (6.51)	0.67 (5.68)	0.02
Influenza vaccine[d]	433 (37.2)	650 (38.6)	0.03	392 (37.8)	393 (37.9)	0.00
Extended pneumococcal vaccines[d]	16 (1.4)	30 (1.8)	0.03	15 (1.4)	16 (1.5)	0.01
**Measures of health care use**						
Number of emergency department visits; mean (SD)[d]	0.65 (1.41)	0.73 (1.57)	0.06	0.66 (1.42)	0.70 (1.54)	0.03
Number of outpatient visits; mean (SD)[d]	5.44 (3.22)	5.62 (3.45)	0.06	5.45 (3.27)	5.41 (3.33)	0.01
Number of hospitalizations; mean (SD)[e]	0.07 (0.27)	0.14 (0.42)	0.20	0.07 (0.28)	0.08 (0.32)	0.02
Hospitalization days for IBD; mean (SD)[e]	0.22 (1.18)	0.69 (3.60)	0.18	0.22 (1.16)	0.22 (1.17)	0.00

[a]All values are reported as number (%) unless otherwise noted
[b]Absolute standardized difference
[c]Assessed on cohort entry
[d]Assessed from 180 days prior to and including the date of cohort entry
[e]Assessed from 180 days to 31 days prior to the date of cohort entry
[f]Assessed from 30 days to 1 day prior to the date of cohort entry
[g]Assessed from 60 days prior to the date of cohort entry
[h]Cumulative miligrams of prednisone equivalent

© 2026 Lyu N et al. *JAMA Network Open.*

eTable 8. Comprehensive Patient Characteristics, Optum Clinformatics Data Mart Database

	Unmatched			Propensity-score Matched		
**Variable**	**Adalimumab (n=607)[a]**	**Infliximab (n=782)[a]**	**ASD[b]**	**Adalimumab (n=496)[a]**	**Infliximab (n=496)[a]**	**ASD[b]**
**Demographic characteristics[c]**						
Year of Cohort Entry Date						
2016	28 (4.6)	55 (7.0)	0.10	28 (5.6)	23 (4.6)	0.05
2017	65 (10.7)	116 (14.8)	0.12	61 (12.3)	63 (12.7)	0.01
2018	87 (14.3)	117 (15.0)	0.02	80 (16.1)	75 (15.1)	0.03
2019	90 (14.8)	90 (11.5)	0.10	67 (13.5)	67 (13.5)	0.00
2020	71 (11.7)	105 (13.4)	0.05	59 (11.9)	62 (12.5)	0.02
2021	78 (12.9)	85 (10.9)	0.06	60 (12.1)	58 (11.7)	0.01
2022	70 (11.5)	89 (11.4)	0.01	61 (12.3)	61 (12.3)	0.00
2023	68 (11.2)	72 (9.2)	0.07	48 (9.7)	58 (11.7)	0.07
2024	50 (8.2)	53 (6.8)	0.05	32 (6.5)	29 (5.8)	0.03
Age; mean (SD)	14.04 (2.58)	13.14 (2.92)	0.33	13.86 (2.68)	13.64 (2.73)	0.08
Age category						
6 - 11	103 (17.0)	222 (28.4)	0.28	99 (20.0)	99 (20.0)	0.00
12 - 17	504 (83.0)	560 (71.6)	0.28	397 (80.0)	397 (80.0)	0.00
Sex						
Male	371 (61.1)	458 (58.6)	0.05	295 (59.5)	299 (60.3)	0.02
Female	236 (38.9)	324 (41.4)	0.05	201 (40.5)	197 (39.7)	0.02
Region						
South	257 (42.3)	285 (36.4)	0.12	201 (40.5)	203 (40.9)	0.01
North Central	189 (31.1)	230 (29.4)	0.04	154 (31.0)	156 (31.5)	0.01
West	94 (15.5)	144 (18.4)	0.08	79 (15.9)	78 (15.7)	0.01
Northeast	66 (10.9)	120 (15.3)	0.13	61 (12.3)	59 (11.9)	0.01
Others[d]	<11	<11	0.04	<11	<11	0.06
**Clinical characteristics[e]**						

© 2026 Lyu N et al. *JAMA Network Open.*

IBD subtype						
Crohn's disease	377 (62.1)	479 (61.3)	0.02	306 (61.7)	307 (61.9)	0.00
Ulcerative colitis	139 (22.9)	154 (19.7)	0.08	106 (21.4)	107 (21.6)	0.01
Unspecified	91 (15.0)	149 (19.1)	0.11	84 (16.9)	82 (16.5)	0.01
IBD disease activity assessment						
C-reactive protein tests ordered	532 (87.6)	730 (93.4)	0.20	453 (91.3)	454 (91.5)	0.01
Colonoscopy/sigmoidoscopy	452 (74.5)	614 (78.5)	0.10	374 (75.4)	373 (75.2)	0.01
Esophagoduodenoscopy	351 (57.8)	451 (57.7)	0.00	289 (58.3)	283 (57.1)	0.02
MR abdomen/ pelvis with contrast	230 (37.9)	356 (45.5)	0.16	199 (40.1)	196 (39.5)	0.01
Gastrointestinal pathogen tests ordered	94 (15.5)	149 (19.1)	0.09	85 (17.1)	82 (16.5)	0.02
CT abdomen/pelvis	80 (13.2)	112 (14.3)	0.03	72 (14.5)	68 (13.7)	0.02
Hospitalization for IBD[f]	14 (2.3)	34 (4.3)	0.11	13 (2.6)	14 (2.8)	0.01
**Baseline Comorbidities[e]**						
Pediatric comorbidity index; mean (SD)	3.80 (2.42)	4.03 (2.53)	0.10	3.91 (2.37)	3.81 (2.53)	0.04
CD With Complications	145 (23.9)	246 (31.5)	0.17	124 (25.0)	132 (26.6)	0.04
UC With Complications	38 (6.3)	57 (7.3)	0.04	33 (6.7)	27 (5.4)	0.05
Abnormal weight loss	208 (34.3)	312 (39.9)	0.12	176 (35.5)	177 (35.7)	0.00
Anemia	182 (30.0)	317 (40.5)	0.22	166 (33.5)	164 (33.1)	0.01
Gastrointestinal bleeding	153 (25.2)	224 (28.6)	0.08	132 (26.6)	126 (25.4)	0.03
Asthma	88 (14.5)	122 (15.6)	0.03	74 (14.9)	73 (14.7)	0.01
Underweight/failure to thrive	65 (10.7)	134 (17.1)	0.19	62 (12.5)	68 (13.7)	0.04
Immunodeficiency	33 (5.4)	35 (4.5)	0.04	23 (4.6)	22 (4.4)	0.01
Protein-calorie Malnutrition	27 (4.4)	65 (8.3)	0.16	26 (5.2)	25 (5.0)	0.01
Obesity/overweight	17 (2.8)	22 (2.8)	0.00	15 (3.0)	13 (2.6)	0.02
Chronic kidney disease[d,e]	<11	<11	-	<11	<11	-
Chronic liver disease[d,e]	<11	<11	0.04	<11	<11	0.02
**Baseline medications**						
Aminosalicylates, previous use[f]	181 (29.8)	187 (23.9)	0.13	130 (26.2)	136 (27.4)	0.03
Aminosalicylates, concurrent use[g]	113 (18.6)	115 (14.7)	0.11	77 (15.5)	82 (16.5)	0.03
Thiopurines, previous use[f]	77 (12.7)	81 (10.4)	0.07	63 (12.7)	58 (11.7)	0.03
Thiopurines, concurrent use[g]	43 (7.1)	51 (6.5)	0.02	36 (7.3)	30 (6.0)	0.05
Methotrexate, previous use[f]	40 (6.6)	38 (4.9)	0.08	31 (6.3)	24 (4.8)	0.00
Methotrexate, concurrent[g]	50 (8.2)	51 (6.5)	0.07	37 (7.5)	37 (7.5)	0.00

© 2026 Lyu N et al. *JAMA Network Open.*

Systemic glucocorticosteroids; mean (SD)[h, i]	521.92 (812.42)	544.49 (811.41)	0.03	544.56 (850.43)	534.24 (743.87)	0.01
Days of Antibiotics; mean (SD)[e]	4.46 (12.93)	5.49 (14.01)	0.08	4.80 (13.78)	4.53 (12.03)	0.02
Days of Antivirals; mean (SD)[e]	0.02 (0.61)	0.03 (0.51)	0.00	0.03 (0.67)	0.02 (0.45)	0.02
Days of antifungals; mean (SD)[e]	0.72 (5.99)	0.65 (4.51)	0.01	0.62 (5.80)	0.76 (5.19)	0.03
Influenza vaccine[e]	234 (38.6)	311 (39.8)	0.03	204 (41.1)	187 (37.7)	0.07
Extended pneumococcal vaccines[e]	<11	<11	0.02	<11	<11	0.02
**Measures of health care use**[e]						
Number of emergency department visit; mean (SD)	0.55 (1.17)	0.74 (1.57)	0.13	0.58 (1.21)	0.58 (1.25)	0.00
Number of outpatient visit; mean (SD)	5.43 (3.02)	5.61 (3.24)	0.06	5.56 (3.08)	5.41 (3.29)	0.05
Number of hospitalizations; mean (SD)	0.09 (0.32)	0.14 (0.45)	0.14	0.10 (0.35)	0.09 (0.36)	0.01
Hospitalization days for IBD; mean (SD)[f]	0.44 (2.26)	0.88 (3.55)	0.15	0.52 (2.48)	0.56 (2.69)	0.02

[a]All values are reported as number (%) unless otherwise noted
[b]Absolute standardized difference
[c]Assessed on cohort entry
[d]Cells containing fewer than 11 patients are masked to protect patient privacy per Optum data use agreement
[e]Assessed from 180 days prior to and including the date of cohort entry
[f]Assessed from 180 days through 31 days prior to the date of cohort entry
[g]Assessed from 30 days to 1 day prior to the date of cohort entry
[h]Assessed from 60 days prior to the date of cohort entry
[i]Cumulative miligrams of prednisone equivalent

© 2026 Lyu N et al. *JAMA Network Open.*

eTable 9. Pooled Infection Component Risks Before and After Propensity Score Matching

	Number of events / total number of patients		
Outcome[a]	**Adalimumab**	**Infliximab (referent)**	**Hazard ratio (95% CI)**
Serious infections			
Acute Meningitis			
Unadjusted	0/1772	0/2467	NA
Propensity score matched	0/1533	0/1533	NA
Acute Osteomyelitis			
Unadjusted	1/1772	1/2467	1.41 (0.09, 22.52)
Propensity score matched	0/1533	1/1533	NA
Bacteremia			
Unadjusted	3/1772	9/2467	0.76 (0.19, 3.02)
Propensity score matched	3/1533	5/1533	1.54 (0.26, 9.22)
Pneumonia			
Unadjusted	0/1772	3/2467	NA
Propensity score matched	3/1533	2/1533	NA
Pyelonephritis			
Unadjusted	0/1772	2/2467	NA
Propensity score matched	0/1533	1/1533	NA
Serious GI Infection			
Unadjusted	6/1772	20/2467	0.60 (0.23, 1.54)
Propensity score matched	3/1533	4/1533	0.69 (0.25, 1.94)
Skin and Soft Tissue			
Unadjusted	16/1772	15/2467	1.46 (0.69, 3.07)
Propensity score matched	15/1533	9/1533	1.70 (0.73, 3.93)
**Outpatient infections**			
Bacterial infections			
Unadjusted	242/1772	313/2467	1.15 (0.97, 1.35)
Propensity score matched	206/1533	198/1533	1.10 (0.90, 1.34)
Lyme			
Unadjusted	0/1772	2/2467	NA
Propensity score matched	0/1533	1/1533	NA

© 2026 Lyu N et al. *JAMA Network Open.*

Mycobacterial			
Unadjusted	0/1772	0/2467	NA
Propensity score matched	0/1533	0/1533	NA
Yeast			
Unadjusted	9/1772	14/2467	1.24 (0.51, 2.99)
Propensity score matched	9/1533	9/1533	1.35 (0.50, 3.62)
Herpes			
Unadjusted	2/1772	4/2467	0.39 (0.04, 3.48)
Propensity score matched	1/1533	2/1533	0.54 (0.05, 5.96)
Influenza			
Unadjusted	31/1772	34/2467	1.34 (0.82, 2.17)
Propensity score matched	21/1533	27/1533	1.05 (0.62, 1.80)
COVID			
Unadjusted	0/1772	3/2467	NA
Propensity score matched	0/1533	2/1533	NA
**Bacterial infections components**			
Acute otitis media			
Unadjusted	55/1772	63/2467	1.30 (0.91,1.86)
Propensity score matched	45/1533	34/1533	1.37 (0.92, 2.03)
Acute sinusitis			
Unadjusted	40/1772	73/2467	0.80 (0.54, 1.18)
Propensity score matched	36/1533	54/1533	0.70 (0.46, 1.08)
Acute bronchitis			
Unadjusted	22/1772	17/2467	1.88 (0.99, 3.57)
Propensity score matched	20/1533	27/1533	1.85 (0.91, 3.76)
Pharyngitis			
Unadjusted	106/1772	125/2467	1.16 (0.90, 1.49)
Propensity score matched	94/1533	86/1533	1.16 (0.86, 1.55)
Pneumonia			
Unadjusted	11/1772	12/2467	1.36 (0.58, 3.16)
Propensity score matched	10/1533	7/1533	1.50 (0.56, 4.00)
UTI			
Unadjusted	12/1772	8/2467	1.00 (0.48, 2.08)

© 2026 Lyu N et al. *JAMA Network Open.*

Propensity score matched	10/1533	8/1533	1.32 (0.50, 3.53)
Skin and soft tissue infections			
Unadjusted	53/1772	61/2467	1.29 (0.90, 1.86)
Propensity score matched	41/1533	36/1533	1.21 (0.77, 1.89)

[a]Measured outcome components were not mutually exclusive.

© 2026 Lyu N et al. *JAMA Network Open.*

eTable 10. Empirically Selected Covariates Included in the High-Dimensional Propensity Score Model

No.	Domain	Code	Description	Frequency
1	Inpatient diagnosis	K51	Ulcerative colitis	At least median frequency
2	Inpatient diagnosis	K51	Ulcerative colitis	At least 1 occurrence
3	Inpatient diagnosis	R10	Abdominal and pelvic pain	At least 1 occurrence
4	Pharmacy (generic name)	—	Budesonide	At least 1 dispensing
5	Outpatient procedure	86160	Complement, antigen, each component	At least Q3 frequency
6	Inpatient diagnosis	R10	Abdominal and pelvic pain	At least median frequency
7	Pharmacy (generic name)	—	Fluocinonide	At least 1 dispensing
8	Inpatient diagnosis	K50	Crohn disease	At least 1 occurrence
9	Inpatient diagnosis	R63	Symptoms and signs concerning food and fluid intake	At least 1 occurrence
10	Inpatient diagnosis	K51	Ulcerative colitis	At least Q3 frequency
11	Outpatient procedure	81001	Urinalysis, automated, with microscopy	At least 1 occurrence
12	Outpatient diagnosis	L30	Other and unspecified dermatitis	At least 1 occurrence
13	Outpatient diagnosis	R45	Symptoms and signs involving emotional state	At least median frequency
14	Outpatient procedure	96413	Chemotherapy/biologic infusion, intravenous, initial up to 1 hour	At least 1 occurrence
15	Inpatient diagnosis	R76	Other abnormal immunological findings in serum	At least 1 occurrence
16	Inpatient diagnosis	E43	Unspecified severe protein-calorie malnutrition	At least 1 occurrence
17	Inpatient diagnosis	K63	Other diseases of intestine	At least 1 occurrence
18	Outpatient procedure	96365	Intravenous infusion, initial up to 1 hour	At least 1 occurrence
19	Outpatient diagnosis	L53	Other erythematous conditions	At least 1 occurrence
20	Outpatient diagnosis	L02	Cutaneous abscess, furuncle, and carbuncle	At least 1 occurrence
21	Outpatient diagnosis	R56	Convulsions, not elsewhere classified	At least median frequency
22	Pharmacy (generic name)	—	Ciprofloxacin hydrochloride	At least 1 dispensing
23	Outpatient diagnosis	R45	Symptoms and signs involving emotional state	At least 1 occurrence
24	Outpatient diagnosis	N83	Noninflammatory disorders of ovary, fallopian tube, and broad ligament	At least Q3 frequency
25	Outpatient diagnosis	Z32	Encounter for pregnancy test and childbirth/pregnancy status incidental	At least median frequency
26	Pharmacy (generic name)	—	Rifaximin	At least 1 dispensing
27	Pharmacy (generic name)	—	Paroxetine hydrochloride	At least median dispensing frequency
28	Summary utilization indicator	—	Total number of outpatient diagnosis codes	Between 0 and Q1
29	Pharmacy (generic name)	—	Budesonide	At least median dispensing frequency
30	Outpatient procedure	99238	Hospital discharge day management, 30 minutes or less	At least 1 occurrence
31	Outpatient diagnosis	M22	Disorder of patella	At least median frequency
32	Inpatient diagnosis	K52	Other and unspecified noninfective gastroenteritis and colitis	At least 1 occurrence
33	Outpatient procedure	96415	Chemotherapy/biologic infusion, intravenous, each additional hour	At least 1 occurrence

© 2026 Lyu N et al. *JAMA Network Open.*

34	Pharmacy (generic name)	—	COVID-19 vaccine, mRNA, BNT162b2 (Pfizer-BioNTech)	At least 1 dispensing/record
35	Outpatient procedure	86800	Thyroglobulin antibody	At least 1 occurrence
36	Outpatient procedure	A0425	Ground mileage, per statute mile	At least 1 occurrence
37	Pharmacy (generic name)	—	Fluticasone propionate/salmeterol xinafoate	At least Q3 dispensing frequency
38	Inpatient diagnosis	K50	Crohn disease	At least median frequency
39	Inpatient diagnosis	F41	Other anxiety disorders	At least 1 occurrence
40	Inpatient diagnosis	D64	Other anemias	At least 1 occurrence
41	Outpatient procedure	96375	Therapeutic/prophylactic/diagnostic injection, each additional sequential IV push	At least 1 occurrence
42	Outpatient procedure	99242	Office consultation for a new or established patient, straightforward/moderate level	At least 1 occurrence
43	Inpatient diagnosis	K21	Gastro-esophageal reflux disease	At least 1 occurrence
44	Outpatient diagnosis	Q79	Congenital malformations of the musculoskeletal system, not elsewhere classified	At least 1 occurrence
45	Outpatient diagnosis	D64	Other anemias	At least 1 occurrence
46	Outpatient procedure	87147	Culture, bacterial; identification by immunologic technique	At least 1 occurrence
47	Outpatient procedure	99053	Services requested between 10:00 PM and 8:00 AM in addition to basic service	At least 1 occurrence
48	Outpatient diagnosis	M99	Biomechanical lesions, not elsewhere classified	At least 1 occurrence
49	Inpatient diagnosis	K59	Other functional intestinal disorders	At least 1 occurrence
50	Outpatient procedure	36415	Collection of venous blood by venipuncture	At least 1 occurrence
51	Outpatient diagnosis	Z32	Encounter for pregnancy test and childbirth/pregnancy status incidental	At least 1 occurrence
52	Outpatient diagnosis	K58	Irritable bowel syndrome	At least Q3 frequency
53	Outpatient procedure	82248	Bilirubin, direct	At least Q3 frequency
54	Outpatient procedure	83615	Lactate dehydrogenase (LDH)	At least 1 occurrence
55	Outpatient procedure	J7030	Infusion, normal saline solution, 1000 mL	At least 1 occurrence
56	Outpatient procedure	92610	Evaluation of oral and pharyngeal swallowing function	At least 1 occurrence
57	Outpatient procedure	31575	Diagnostic laryngoscopy, flexible fiberoptic	At least 1 occurrence
58	Outpatient diagnosis	I89	Other noninfective disorders of lymphatic vessels and lymph nodes	At least 1 occurrence
59	Outpatient diagnosis	H72	Perforation of tympanic membrane	At least 1 occurrence
60	Pharmacy (generic name)	—	Metronidazole	At least 1 dispensing
61	Outpatient procedure	G0378	Hospital observation service, per hour	At least Q3 frequency
62	Outpatient diagnosis	R30	Pain associated with micturition	At least 1 occurrence
63	Summary utilization indicator	—	Total number of dispensed medications identified by generic name	Between Q3 and maximum
64	Inpatient diagnosis	R19	Other symptoms and signs involving the digestive system and abdomen	At least 1 occurrence
65	Outpatient procedure	86682	Antibody test, helminth	At least 1 occurrence
66	Outpatient procedure	96413	Chemotherapy/biologic infusion, intravenous, initial up to 1 hour	At least median frequency
67	Pharmacy (generic name)	—	Rizatriptan benzoate	At least 1 dispensing
68	Outpatient diagnosis	Z23	Encounter for immunization	At least 1 occurrence

© 2026 Lyu N et al. *JAMA Network Open.*

69	Outpatient diagnosis	F81	Developmental disorders of scholastic skills	At least median frequency
70	Outpatient procedure	J2920	Methylprednisolone sodium succinate, 100 mg	At least 1 occurrence
71	Outpatient procedure	87426	Infectious agent antigen detection by immunoassay technique, SARS-CoV-2	At least 1 occurrence
72	Outpatient diagnosis	M35	Other systemic involvement of connective tissue	At least 1 occurrence
73	Outpatient diagnosis	B35	Dermatophytosis	At least 1 occurrence
74	Inpatient diagnosis	R00	Abnormalities of heart beat	At least 1 occurrence
75	Outpatient diagnosis	M54	Dorsalgia	At least 1 occurrence
76	Outpatient procedure	85652	Erythrocyte sedimentation rate	At least median frequency
77	Inpatient diagnosis	K92	Other diseases of digestive system	At least 1 occurrence
78	Outpatient diagnosis	M25	Other joint disorder, not elsewhere classified	At least 1 occurrence
79	Summary utilization indicator	—	Total number of unique outpatient diagnosis codes	Between Q3 and maximum
80	Outpatient procedure	99232	Subsequent hospital inpatient care	At least 1 occurrence
81	Outpatient procedure	96374	Therapeutic/prophylactic/diagnostic injection, intravenous push, single or initial substance	At least 1 occurrence
82	Outpatient diagnosis	M22	Disorder of patella	At least 1 occurrence
83	Inpatient diagnosis	E86	Volume depletion	At least 1 occurrence
84	Outpatient procedure	0124A	COVID-19 vaccine, booster dose/administration code	At least 1 occurrence
85	Outpatient diagnosis	M12	Other specific arthropathies	At least 1 occurrence
86	Outpatient diagnosis	K90	Intestinal malabsorption	At least 1 occurrence
87	Outpatient procedure	71020	Chest radiograph, 2 views	At least 1 occurrence
88	Outpatient diagnosis	Z68	Body mass index (BMI)	At least median frequency
89	Inpatient diagnosis	Z20	Contact with and suspected exposure to communicable diseases	At least 1 occurrence
90	Summary utilization indicator	—	Total number of dispensed medications identified by generic name	Between 0 and Q1
91	Outpatient diagnosis	Z92	Personal history of medical treatment	At least median frequency
92	Summary utilization indicator	—	Total number of unique dispensed medications identified by generic name	Between Q3 and maximum
93	Inpatient diagnosis	D63	Anemia in chronic diseases classified elsewhere	At least 1 occurrence
94	Outpatient procedure	98941	Chiropractic manipulative treatment, 3 to 4 regions	At least 1 occurrence
95	Outpatient procedure	99393	Preventive medicine service, established patient, late childhood	At least 1 occurrence
96	Outpatient diagnosis	A49	Bacterial infection of unspecified site	At least 1 occurrence
97	Outpatient procedure	87430	Infectious agent antigen detection by immunoassay technique, Streptococcus, group A	At least 1 occurrence
98	Outpatient procedure	84450	Aspartate aminotransferase (AST)	At least 1 occurrence
99	Outpatient diagnosis	L92	Granulomatous disorders of skin and subcutaneous tissue	At least 1 occurrence
100	Summary utilization indicator	—	Total number of inpatient diagnosis codes	Between Q3 and maximum
101	Outpatient procedure	96361	Intravenous infusion, hydration, each additional hour	At least 1 occurrence

© 2026 Lyu N et al. *JAMA Network Open.*

102	Pharmacy (generic name)	—	Guanfacine hydrochloride	At least median dispensing frequency
103	Outpatient diagnosis	R31	Hematuria	At least median frequency
104	Outpatient procedure	76705	Ultrasound, abdominal, limited	At least median frequency
105	Outpatient diagnosis	R82	Other abnormal findings in urine	At least median frequency
106	Outpatient diagnosis	N30	Cystitis	At least median frequency
107	Outpatient diagnosis	D48	Neoplasm of uncertain or unknown behavior	At least Q3 frequency
108	Inpatient diagnosis	M25	Other joint disorder, not elsewhere classified	At least 1 occurrence
109	Outpatient procedure	J3420	Vitamin B12 injection, up to 1000 mcg	At least 1 occurrence
110	Outpatient procedure	99224	Subsequent observation care	At least 1 occurrence
111	Outpatient procedure	83020	Hemoglobin electrophoresis	At least Q3 frequency
112	Outpatient diagnosis	S13	Dislocation and sprain of joints and ligaments at neck level	At least 1 occurrence
113	Outpatient diagnosis	R85	Abnormal findings in specimens from digestive organs and abdominal cavity	At least median frequency
114	Outpatient diagnosis	N85	Other noninflammatory disorders of uterus, except cervix	At least 1 occurrence
115	Outpatient diagnosis	K38	Other diseases of appendix	At least 1 occurrence
116	Outpatient diagnosis	K38	Other diseases of appendix	At least median frequency
117	Inpatient diagnosis	J84	Other interstitial pulmonary diseases	At least 1 occurrence
118	Pharmacy (generic name)	—	Norethindrone-ethinyl estradiol	At least 1 dispensing
119	Pharmacy (generic name)	—	Methotrexate	At least median dispensing frequency
120	Pharmacy (generic name)	—	Desmopressin acetate	At least median dispensing frequency
121	Outpatient procedure	96372	Therapeutic/prophylactic/diagnostic injection, subcutaneous or intramuscular	At least Q3 frequency
122	Outpatient diagnosis	M25	Other joint disorder, not elsewhere classified	At least median frequency
123	Outpatient procedure	J7050	Infusion, normal saline solution, 250 mL	At least 1 occurrence
124	Outpatient procedure	90744	Hepatitis B vaccine, pediatric/adolescent dosage	At least 1 occurrence
125	Outpatient procedure	96415	Chemotherapy/biologic infusion, intravenous, each additional hour	At least median frequency
126	Outpatient procedure	72197	Magnetic resonance imaging, pelvis, with and without contrast	At least 1 occurrence
127	Inpatient diagnosis	K62	Other diseases of anus and rectum	At least 1 occurrence
128	Outpatient procedure	46060	Incision and drainage of ischiorectal and/or perirectal abscess	At least 1 occurrence
129	Outpatient diagnosis	R56	Convulsions, not elsewhere classified	At least Q3 frequency
130	Outpatient diagnosis	E04	Other nontoxic goiter	At least 1 occurrence
131	Outpatient diagnosis	D80	Immunodeficiency with predominantly antibody defects	At least median frequency
132	Outpatient diagnosis	R93	Abnormal findings on diagnostic imaging	At least Q3 frequency
133	Outpatient procedure	99221	Initial hospital inpatient care	At least 1 occurrence
134	Outpatient procedure	90836	Psychotherapy, 45 minutes with evaluation and management service	At least median frequency
135	Outpatient diagnosis	R41	Other symptoms and signs involving cognitive functions and awareness	At least Q3 frequency
136	Outpatient diagnosis	F81	Developmental disorders of scholastic skills	At least Q3 frequency
137	Inpatient diagnosis	Z90	Acquired absence of organs, not elsewhere classified	At least 1 occurrence

© 2026 Lyu N et al. *JAMA Network Open.*

138	Inpatient diagnosis	J30	Vasomotor and allergic rhinitis	At least 1 occurrence
139	Outpatient diagnosis	K21	Gastro-esophageal reflux disease	At least Q3 frequency
140	Outpatient diagnosis	R10	Abdominal and pelvic pain	At least 1 occurrence
141	Outpatient diagnosis	D48	Neoplasm of uncertain or unknown behavior	At least 1 occurrence
142	Outpatient procedure	A0427	Ambulance service, advanced life support, emergency transport	At least 1 occurrence
143	Outpatient procedure	87086	Urine culture, quantitative colony count	At least 1 occurrence
144	Outpatient procedure	81025	Urine pregnancy test	At least 1 occurrence
145	Outpatient diagnosis	L73	Other follicular disorders	At least Q3 frequency
146	Pharmacy (generic name)	—	Celecoxib	At least 1 dispensing
147	Outpatient procedure	86885	Antihuman globulin test, indirect, each serum technique	At least 1 occurrence
148	Outpatient diagnosis	Z53	Persons encountering health services for specific procedures, not carried out	At least median frequency
149	Outpatient diagnosis	E87	Other disorders of fluid, electrolyte, and acid-base balance	At least Q3 frequency
150	Pharmacy (generic name)	—	Potassium chloride	At least 1 dispensing

Footnote:

hdPS indicates high-dimensional propensity score. Covariates were empirically selected from inpatient diagnosis, outpatient diagnosis, outpatient procedure, pharmacy dispensing, and summary utilization dimensions during baseline. Pharmacy covariates were identified by generic drug name. For hdPS-selected covariates, thresholds such as “at least 1,” “at least median frequency,” and “at least Q3 frequency” indicate whether the frequency of a given code or medication during baseline met or exceeded 1 occurrence, the median, or the third quartile among patients with at least 1 occurrence.

© 2026 Lyu N et al. *JAMA Network Open.*

eTable 11. Pooled Negative Control Outcome Before and After Propensity Score Matching

	No. / total No. (%)			
**Outcome**	**Infliximab (referent)**	**Adalimumab**	**Difference (95% CI) (%)**	**Hazard ratio (95% CI)**
**Tendonitis/tendinopathy**				
Unadjusted	13/2467 (0.5)	14/1772 (0.8)	0.26 (0.25, 0.27)	1.55 (0.72, 3.34)
PS-matched	10/1533 (0.7)	12/1533 (0.8)	0.13 (0.12, 0.14)	1.26 (0.55, 2.91)

© 2026 Lyu N et al. *JAMA Network Open.*
