# Supplementary material for: Risk of Infections With Infliximab vs Adalimumab Among Children With Inflammatory Bowel Disease
Source: JAMA Netw Open. 2026 Jul 10;9(7):e2622684. doi: 10.1001/jamanetworkopen.2026.22684 (PMC13355144; doi:10.1001/jamanetworkopen.2026.22684)
Supplement: Supplement 2. — Data Sharing Statement [file jamanetwopen-e2622684-s002.pdf]

Data Sharing Statement

Lyu. Risk of Infections With Infliximab vs Adalimumab Among Children With Inflammatory Bowel Disease. *JAMA Netw Open*. Published July 10, 2026.
doi:10.1001/jamanetworkopen.2026.22684

Data
Data available: No
